# Decoding neoantigen-encoding tumor-specific transcripts unveils a shared target reservoir for immunotherapy in hepatocellular carcinoma

**DOI:** 10.1136/jitc-2026-015428

**Published:** 2026-07-21

**Authors:** Peng Lin, Yifan Wen, Jingjing Zhao, Feifei Zhang, Yaoming Su, Huiyi He, Hongwu Yu, Qiaojuan Li, Chengye Liu, Zhixiang Hu, Yan Li, Zhuting Fang, Linhui Liang, Shenglin Huang

**Affiliations:** 1 Department of Integrative Oncology, Fudan University Shanghai Cancer Center, and Shanghai Key Laboratory of Medical Epigenetics, Institutes of Biomedical Sciences, Fudan University, Shanghai, China; 2 Department of Oncology, Fudan University, Shanghai, China; 3 Department of Oncology and Vascular Interventional Therapy, Clinical Oncology School of Fujian Medical University, Fujian Cancer Hospital (Fujian Branch of Fudan University Shanghai Cancer Center), Fuzhou, China; 4 State Key Laboratory of Genetics and Development of Complex Phenotypes, Fudan University, Shanghai, Shanghai, China

**Keywords:** Immunotherapy, Human leukocyte antigen - HLA, Hepatocellular Carcinoma

## Abstract

**Background:**

Primary liver cancer, predominantly hepatocellular carcinoma (HCC), has limited therapeutic options. While mutation-derived neoantigen vaccine holds promise, its success is hindered by low antigen availability. This study explores transcriptome-derived neoantigens (neoantigen-encoding tumor-specific transcripts, neoTSTs) in HCC, characterizing their features, generation mechanisms, and therapeutic potential.

**Methods:**

We developed a computational pipeline integrating STAR/StringTie-based transcript assembly with multiexon/single-exon reference datasets (23,972 human control samples) for tumor-specific transcripts (TSTs) identification. A custom sliding-window algorithm compared TST-encoded peptides against UniProt, with neoTSTs predicted using netMHCPan. This framework was applied to 1,013 patients with liver cancer. NeoTSTs were validated through proteomics, immunopeptidomics, and HLA-transgenic models. Multiomics analyses characterized splicing patterns, transposable elements, and transcription factor regulation. Single-cell RNA-seq and Hep53.4 murine models assessed tumor coverage and immunotherapeutic efficacy.

**Results:**

We analyzed RNA-seq data from 1,013 patients with liver cancer and constructed a multilayered reference dataset. Using a customized pipeline, we identified an average of 60 neoTSTs per patient, significantly surpassing mutation-derived neoantigens (neoMuts). NeoTSTs exhibited higher population frequencies, with 73.1% providing multiple epitopes, and were validated through mass spectrometry and HLA transgenic mouse models. Mechanistically, neoTSTs were generated via retained introns, transposable element activation, HNF4A-regulated alternative promoters, and de novo transmembrane domain generation. Single-cell analysis revealed neoTSTs cover >75% of tumor cells and identified antigen-presenting cancer-associated fibroblasts that enriched in immunotherapy responders and amplified CD4^+^ T-cell responses. In murine HCC models, neoTST vaccination outperformed neoMuts, inducing dual major histocompatibility complex-I/II activation and significant tumor growth inhibition.

**Conclusions:**

NeoTSTs represent a superior neoantigen source in HCC, compensating for the limitations of mutation-derived targets. The remarkable abundance and patient-to-patient sharedness of neoTSTs underscore their dual potential: (1) as personalized immunotherapeutic targets, and (2) as broadly applicable antigens for low-TMB tumors. These findings provide a transformative framework for expanding treatment options in HCC immunotherapy.

**WHAT IS ALREADY KNOWN ON THIS TOPIC**

- Hepatocellular carcinoma (HCC) has a poor prognosis, and immunotherapy outcomes remain limited. While neoantigen vaccines show promise, current research largely focuses on mutation-derived neoantigens, which are scarce in low-mutation tumors like HCC. Existing methods for identifying transcriptional neoantigens face challenges such as incomplete datasets and inconsistent criteria.

**WHAT THIS STUDY ADDS**

- Analysis of 1013 liver cancer transcriptomes revealed abundant neoantigen-encoding tumor-specific transcripts. These neoantigens arise via intron retention, transposable element activation, and HNF4A-mediated regulation. Additionally, antigen-presenting cancer-associated fibroblasts were found to enhance CD4^+^ T-cell activation and immunotherapy response.

**HOW THIS STUDY MIGHT AFFECT RESEARCH, PRACTICE OR POLICY**

- Our work expands the target repertoire for HCC immunotherapy and provides a standardized analytical framework. The findings support the clinical translation of transcriptional neoantigen-based strategies and deepen the mechanistic understanding of neoantigen generation and function.

## Background

 Primary liver cancer constitutes the third leading cause of cancer-related mortality globally. Hepatocellular carcinoma (HCC), representing approximately 90% of hepatic malignancies, is predominantly diagnosed at advanced stages, conferring poor prognosis.^1 2^ Current standard therapies-including surgical resection, liver transplantation, local ablation, and chemotherapy-exhibit significant limitations: chemotherapy demonstrates restricted efficacy with substantial toxicity, while postoperative recurrence occurs in ~70% of resected patients and 20% of transplant recipients within 5 years.^3^ For advanced disease, first-line systemic agents (sorafenib, lenvatinib) provide limited clinical benefit.^4–6^ Notably, recent years have witnessed remarkable advancements in immunotherapy for HCC management.^7^

Current advancements in cancer immunotherapy have primarily focused on immune checkpoint inhibitors (ICIs),^8^ with multiple clinical trials demonstrating their efficacy as first-line treatments for advanced HCC.^9 10^ However, broader application of ICIs faces significant challenges in HCC, particularly among patients with immunosuppressive tumor phenotypes.^11–13^ Most patients derive limited clinical benefit due to insufficient tumor-infiltrating lymphocytes (TILs). Beyond ICIs, tumor-specific neoantigens have emerged as promising targets for cancer immunotherapy.^14–17^ These uniquely tumor-expressed antigens elicit authentic tumor-specific T-cell responses,^18 19^ induce durable immunity, and minimize therapy-related autoimmunity risks.^15^ Critically, neoantigen vaccines simultaneously engage CD4^+^ helper T cells and CD8^+^ cytotoxic T cells, reprogramming the tumor microenvironment to convert immunologically “cold” tumors into “hot” ones-thereby overcoming primary resistance to ICIs.^20^ Additionally, clinical studies have demonstrated the potential of neoantigen vaccines in melanoma, non-small cell lung cancer, and renal cell carcinoma.^21–23^ Notably, recent research has shown that messenger RNA (mRNA)-based neoantigen vaccines derived from somatic mutations can induce long-term T-cell activation in pancreatic ductal adenocarcinoma (PDAC).^24 25^ Recent advances in HCC neoantigen vaccine research were demonstrated by a Johns Hopkins University School of Medicine clinical trial showing that personalized tumor neoantigen vaccines can potentiate programmed cell death protein-1 (PD-1) inhibitor responses in patients with HCC.^23^ However, most neoantigen vaccines in these studies are mutation-derived, and such neoantigens typically arise from low-frequency somatic mutations, which are rarely shared among patients due to tumor heterogeneity. Moreover, tumors with low mutational burden (eg, HCC and PDAC) often lack sufficient mutation-derived neoantigen (neoMut) targets, potentially limiting the efficacy of such therapies.^26 27^ These findings highlight the limitations of relying solely on neoMuts in HCC and underscore the urgent need for alternative approaches, such as transcriptome-derived neoantigen discovery, to expand the repertoire of actionable targets.

Recent advances in cancer immunology have revealed that transcriptome-derived neoantigens play a pivotal role in eliciting potent antitumor immune responses.^28^ Aberrant RNA splicing generates highly immunogenic neoantigens with demonstrated antitumor efficacy in preclinical models.^29^ These splicing-derived epitopes offer enhanced stability and predictability advantages.^30^ Our Assembling Splice Junctions Analysis (ASJA) algorithm^31 32^ has identified clinically relevant tumor-specific transcripts (TSTs) like LIN28B-TST in HCC and macrophage receptor with collagenous structure-TST (MARCO-TST) in triple-negative breast cancer.^33 34^ Pancancer analyses have confirmed TSTs as a promising neoantigen source.^27^ However, the high complexity of the transcriptome poses significant challenges for validating the specificity of neoantigens. Despite advances in reference datasets, HCC’s transcriptome-originated neoantigens landscape remains underexplored.

To comprehensively identify transcript-derived neoantigens in liver cancer, we conducted a systematic analysis of RNA sequencing (RNA-seq) data from 1,013 patients with liver cancer, including 824 HCC, 65 intrahepatic cholangiocarcinomas (ICCs), and 124 hepatoblastomas (HBs). We established a multilayered reference dataset comprising 22,734 normal tissue samples, 459 adjacent non-tumor tissues, and 779 non-cancerous liver disease samples. Using a customized RNA-seq-based neoantigen identification pipeline with stringent thresholds for multialignment analysis, we systematically identified TSTs (including single-exonic and multiexonic transcripts) and their corresponding neoantigens. Our analysis revealed an average of 60 neoantigen-encoding TSTs (neoTSTs) per patient, demonstrating significantly greater abundance and coverage compared with neoMuts. Using HLA-A*02:01 and HLA-A*11:01 transgenic mouse models, we validated neoTST-specific CD8^+^ T cell activation. We found that neoTSTs arose from retained introns, transposable element (TE) activation, HNF4A-regulated promoters, and de novo transmembrane domain (TMD) generation. Furthermore, we discovered that antigen-presenting cancer-associated fibroblasts (apCAFs) can activate CD4^+^ helper T cells through neoTST presentation, suggesting a potential therapeutic target for combination immunotherapy. Therapeutic evaluation in murine HCC model demonstrated significant tumor growth inhibition following neoTST vaccination, highlighting its clinical potential.

## Methods

### Integration of multicenter liver cancer RNA-seq data

To comprehensively characterize the transcriptomic landscape of liver cancer, we integrated RNA-seq data from 1,013 liver tissue samples, including HCC (n=824), HB (n=124), and ICC (n=65). The data were aggregated from multiple public repositories and in-house sequencing efforts to ensure broad representation of molecular subtypes and clinical contexts. For HCC, 373 samples were obtained as Binary Alignment/Map (BAM) files from The Cancer Genome Atlas Program (TCGA, https://www.cancer.gov/ccg/research/genome-sequencing/tcga), 105 samples were sequenced internally (raw FASTQ files), and the remaining samples were compiled from nine independent Gene Expression Omnibus (GEO, https://www.ncbi.nlm.nih.gov/geo) datasets. All 124 HB samples were curated from seven GEO datasets, while ICC samples included 65 samples from four GEO datasets.

For non-TCGA samples, raw FASTQ files were processed using STAR (V.2.5.3a)^35^ for alignment to the GRCh38/hg38 p12 reference genome in two-pass mode with chimeric junction detection enabled, followed by duplicate marking with Picard Tools (V.2.23.3, https://broadinstitute.github.io/picard/). TCGA BAM files were converted to FASTQ using samtools bam2fq and reprocessed identically to ensure uniformity. Transcript abundance was quantified via StringTie (V.2.2.1)^36^ with GENCODE V.29 annotations. This standardized pipeline enabled robust cross-cohort comparisons of liver cancer subtypes.

### Reference transcriptome dataset construction

To establish a comprehensive reference transcriptome dataset for comparative analysis, we integrated RNA-seq data from over 20,000 samples, encompassing normal tissues, adjacent non-tumor tissues, and disease-associated liver tissues across 30 distinct tissue types. The dataset included 1,013 liver tissue samples, 459 adjacent non-tumor samples (of which 50 were paired with tumor samples from our internal cohort), and 22,734 samples from other normal tissues, primarily sourced from the Genotype-Tissue Expression (GTEx V.8) project, TCGA, and GEO databases. To account for potential confounding effects of non-neoplastic liver diseases, we supplemented the reference with 779 RNA-seq profiles from non-cancer disease tissues (eg, cirrhosis, hepatitis B/C), curated from 11 independent GEO datasets, 1 ArrayExpress (https://www.ebi.ac.uk/arrayexpress/) dataset: E-MTAB-6863 and 1 in-house data. This diverse dataset ensures robust background signals for tumor-specific analyses while controlling for inflammation-related and fibrosis-related transcriptional changes. All data were uniformly processed using the pipeline described in the integration of multicenter liver cancer RNA-seq data section.

All tissue RNA-seq data were uniformly processed using StringTie for transcript assembly and quantification. Alternative splicing events were identified with ASJA,^31^ and expression levels were quantified as coverage per 10 million reads (CPT). Subsequently, we constructed a comprehensive reference tissue database by generating 33 independent reference cohorts for each exon junction and single-exon transcript, derived from 30 normal tissue types, adjacent non-tumor tissues, and 3 liver disease sources. For each single-exon transcript, a unique ID was confirmed based on their open reading frame (ORF). For each cohort, three key parameters were calculated: (1) median expression (CPT/transcripts per million), (2) detection frequency, and (3) maximum expression value. This multidimensional reference framework enables robust normalization and context-specific analysis of splicing-derived neoantigens.

### TST-derived neoantigens prediction module

To predict TST-derived neoantigens, single-exon and multiexon TSTs were first merged and filtered based on coding potential, as determined by CPAT (V.0.1).^37^ Only those transcripts classified as coding by CPAT and containing a complete ORF were retained as coding TSTs. To minimize false positives, only ORFs of coding TSTs directly resulting from tumor-specific transcriptional events (tumor-specific splicing) and single-exon TSTs were translated into protein sequences (in silico translation) by Biopython (V.1.81).

20,420 protein sequences were downloaded from UniProt^38^ as reference protein library. By comparing with the reference protein, the newly identified peptide segments encoded by each transcript were confirmed. For each new peptide segment, 11 amino acids were added at both its beginning and end, resulting in the candidate peptide segment used for predicting potential antigenic epitopes. If the new peptide is at the N-terminus or C-terminus of the protein, then only extend one side of it. HLA class I genotyping was performed using arcasHLA (V.3.9)^39^ for samples from GEO and in-house data. HLA typing results for TCGA-LIHC (Liver Hepatocellular Carcinoma) samples were obtained from the Genomic Data Commons (GDC) (see Prediction HLA typing section). Only HLA alleles supported by NetMHCpan (V.4.1)^40^ were retained. In total, high-confidence HLA genotypes were determined for 1,048 samples.

NetMHCpan was used to predict 8–12 amino acid peptides binding to HLA, using the parameters “-f -inptype 0 -BA -xls -a”. Peptides with predicted binding affinity scores (IC50) <500 nM were classified as either strong binders or weak binders and considered candidate neoantigens. Final TST-derived neoantigens were defined as peptides absent in reference protein library.

### Animal model and experimental design

6–8 weeks old male C57BL/6 mice (Shanghai Jihui Laboratory Animal Care) and genetically modified strains (B6-hHLAA11.1/hB2M and B6-hHLAA2.1/hB2M; GemPharmatech) were used in this study. To establish Hep-53.4 subcutaneous tumors, 2.5×10⁵ cells suspended in 200 µL serum-free DMEM were injected into the right dorsal flank. On day 3 postinoculation, mice were randomly allocated to three treatment groups: (1) Control: 100 µL phosphate-buffered saline administered intramuscularly (i.m.) every 7 days (two doses total); (2) neoTSTs: 5 µg neoTST mRNA-LNP (Lipid Nanoparticles) (i.m., 100 µL) every 7 days (two doses); (3) neoMuts: 5 µg neoMut mRNA-LNP (i.m., 100 µL) every 7 days (two doses). Tumor dimensions were measured every 2–3 days using digital calipers, with volumes calculated as V = (L×W²)/2. A strict endpoint criterion (tumor volume >1,500 mm³ or 20 mm in any dimension) was enforced. Mice were housed under specific pathogen-free conditions (12 hours light/dark cycle, 20–22°C, 40–60% humidity). All procedures complied with Shanghai Laboratory Animal Care Association guidelines and were approved by the Fudan University Institutional Animal Care and Use Committee (IACUC protocol: 202510FD0002).

### Statistical analyses

Statistical analyses were performed using R (V.4.0.2) and Python (Python V.3.9.18). Comparisons between two groups were assessed by the Wilcoxon rank-sum test. Survival analyses used Kaplan-Meier estimators with log-rank tests to determine statistical significance. One-way analysis of variance was employed for group comparisons in tumor size and flow cytometry data; results are presented as mean±SEM; Significance levels were defined as follows: *p<0.05, **p<0.01, ***p<0.001, ****p<0.0001.

Full and detailed descriptions of research materials and experimental methods are provided in online supplemental information.

## Results

### Landscape of neoantigen-encoding tumor-specific transcripts in liver cancer

**Figure 1:**
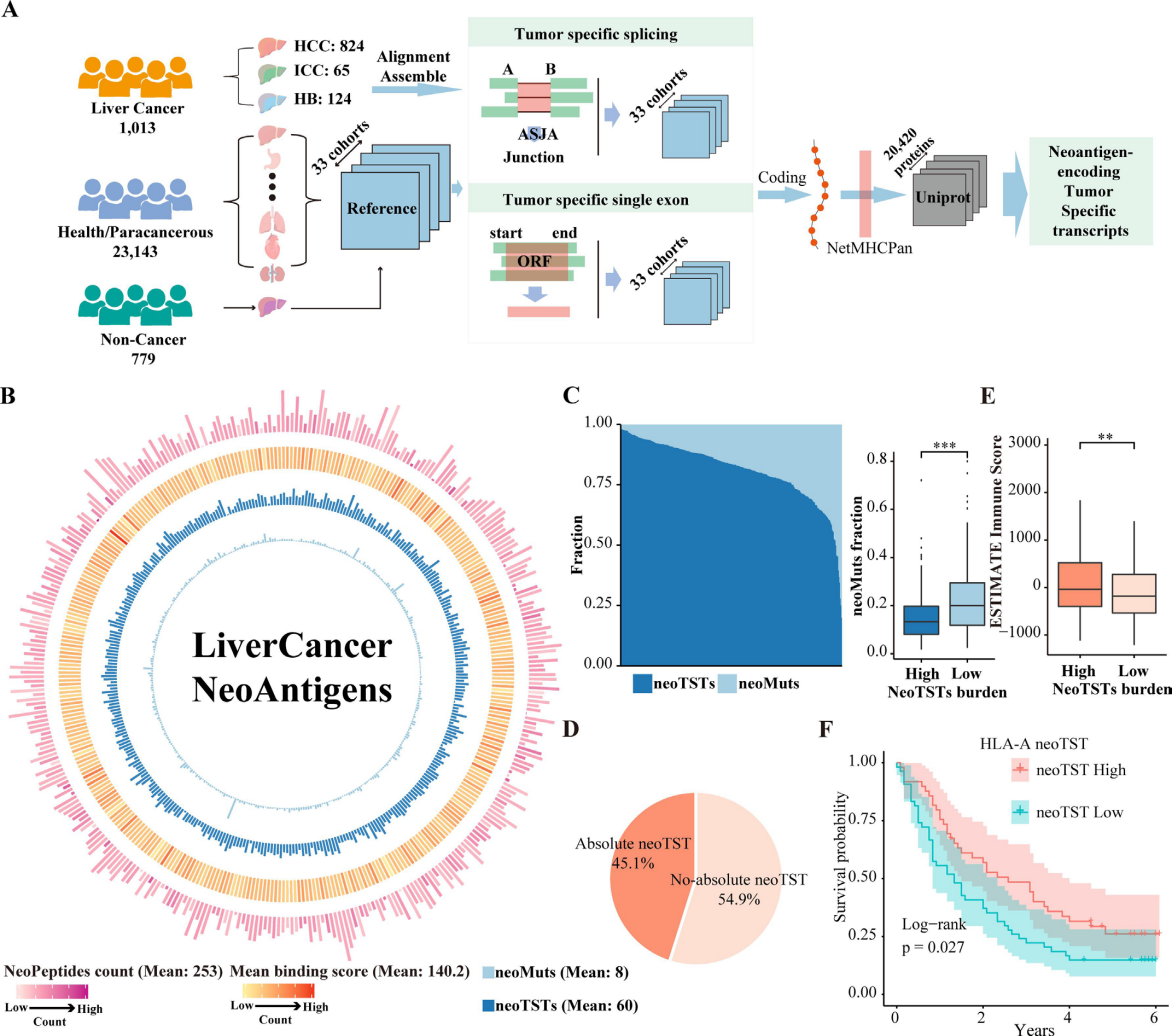
Landscape of neoTSTs in 1,013 liver cancer. (**A**) Workflow for identifying neoTSTs: (1) multicenter data collection and construction of reference datasets; (2) detected TSTs separately for single-exon and multiexon transcripts; (3) neoantigen prediction from coding TSTs. (**B**) Circos plot summarizing neoantigen features per liver cancer sample: (1) number of neoTST-derived neoantigens, the height of the columns and the intensity of the colors indicate the quantity of neoPeptides. The taller the column and the darker the color, the greater the quantity; (2) binding affinity scores of TST-derived neoantigens; (3) number of neoTSTs; and (4) number of neoMuts. (**C**) Distribution and comparative analysis of neoTST and neoMut burden in patients. Left panel: Stacked bar plot displaying the relative proportion of neoTSTs (dark blue) and neoMuts (light blue) for each individual patient. Right panel: Boxplot comparing neoMut proportions between high versus low neoTST-burden group. Statistical significance was determined by Wilcoxon rank-sum test (***p<0.001). (**D**) The pie chart shows the proportions of absolute and non-absolute neoTSTs. (**E**) The boxplot demonstrates significantly higher immune scores (calculated via ESTIMATE^59^ algorithm) in patients with high neoTST burden compared with those with low neoTST burden. Statistical significance was determined by Wilcoxon rank-sum test (**p<0.01). (**F**) Kaplan-Meier survival analysis stratified by neoTST burden (high vs low) of HLA-A in in-house cohort. Log-rank test was used. The average value of the neoTSTs burden was used as the cut-off point. ASJA, Assembling Splice Junctions Analysis; ESTIMATE, Estimation of STromal and Immune cells in MAlignant Tumor tissues; HB, hepatoblastoma; HCC, hepatocellular carcinoma; ICC, intrahepatic cholangiocarcinoma; neoMuts; mutation-derived neoantigens; neoTSTs, neoantigen-encoding tumor-specific transcripts; ORF, open reading frame; TSTs, tumor-specific transcripts.

RNA-seq data from 1,013 liver cancer samples were analyzed to comprehensively characterize transcript-derived neoantigens in liver cancer (figure 1A, online supplemental figure S1A). To rigorously identify TSTs, we constructed a comprehensive reference dataset consisting of 33 independent reference cohorts, which included over 20,000 normal tissue samples (29 tissues), 459 adjacent non-tumor tissues, and 779 non-cancer liver disease samples, encompassing nearly 3 million splice junctions (figure 1A, online supplemental figure S1A,B). We developed a robust computational pipeline for neoantigen discovery (figure 1A, Methods): (1) Transcript assembly: RNA-seq data from tumor and control samples were aligned using STAR and assembled into transcripts with StringTie. (2) Reference construction: Comprehensive reference datasets were built for both multiexonic and single-exonic transcripts. (3) TST detection: Tumor samples were compared against controls using stringent thresholds to identify TSTs under multiexonic and single-exonic conditions. (4) Neoantigen prediction and evaluation: ORFs were predicted computationally to assess coding potential and identify novel peptides. Potential epitopes were predicted using NetMHCpan (V.4.1) with affinity evaluation. This integrated pipeline enables systematic identification of neoTSTs and their potential epitopes in liver cancer.

Approximately 150,000 splice junctions were detected per patient by ASJA (online supplemental figure S1C). Through stringent filtering criteria, we identified an average of 300 TSTs per tumor sample, which were further refined to ~60 neoTSTs per case (44 multiexonic transcripts and 16 single-exonic transcripts; figure 1B, online supplemental figure S1D,E). Among these, 45.1% of all identified neoTSTs are absolute (completely absent in normal tissues) (figure 1D), while 54.9% are non-absolute (ie, expressed at minimal levels in a small subset of normal samples). In stark contrast, only eight neoMuts were identified per patient on average, highlighting the quantitative superiority of neoTSTs (online supplemental figure S1F). Considering the potential of neoTSTs as therapeutic targets, stable and continuous expression in tumor cells is essential. Beyond applying stringent expression and frequency filters, we analyzed the expression of all neoTSTs in different tumor stages and found that they were stably expressed in different stages of cancer (online supplemental figure S1G), reinforcing the persistent nature of these identified transcripts. Immunogenicity prediction revealed that each neoTST generated ~5 presentable peptides (253 NeoPeptides/patient on average) with a mean HLA-I affinity score of 140.2, demonstrating high binding potential (online supplemental figure S1H). Notably, neoTST and neoMut burdens exhibited a complementary pattern: patients with low neoMuts loads showed significantly elevated neoTST levels (figure 1C), suggesting that neoTSTs effectively compensate for the paucity of presented epitopes in TMB-low tumors.

Comparative analysis revealed that the number of candidated neoTSTs identified was remarkably consistent across the three cancer types (online supplemental figure S1I). Despite substantial interpatient and intertumor heterogeneity, neoTSTs exhibited significant overlap between cancer types, with 24.6% of HB-associated neoTSTs and 27.5% of ICC-associated neoTSTs being shared with HCC (online supplemental figure S1J). Given HCC’s predominance among liver cancers, subsequent analyses focus specifically on this malignancy. Our analysis of 824 HCC cases identified 347 patients (42.1%) with detectable hepatitis B virus (HBV) infection (online supplemental figure S1K). Comparative analysis demonstrated that HBV-positive patients exhibited a significantly enriched repertoire of transcript-derived neoantigens (p<0.05) (online supplemental figure S1L). These findings suggest that HBV infection may actively promote neoantigen generation through the formation of neoTSTs, potentially via virus-induced genomic instability or transcriptional dysregulation.

We evaluated the immune activation score for each patient and found that those with a high neoTST burden exhibited significantly higher immune scores, suggesting stronger immune activation (figure 1E). Given that immune activation levels are often associated with improved clinical outcomes, we further analyzed the survival correlation of neoPeptide burden across different HLA subtypes, accounting for population heterogeneity and HLA preferences. While the overall neoTST burden showed no clear prognostic association, distinct patterns emerged in specific populations: in European/American cohorts, patients with high HLA-C neoPeptide loads demonstrated significantly better survival outcomes, whereas in Asian cohorts, HLA-A emerged as the dominant prognostic marker (figure 1F, online supplemental figure S1M,N). In contrast, neoMuts showed no significant correlation with patient prognosis (online supplemental figure S1O). These findings highlight population-specific immunological heterogeneity and suggest that HLA-restricted neoTST presentation may serve as a more robust biomarker for immune responsiveness than conventional mutation-based neoantigens.

### Mass spectrometry and immunopeptidomics-based identification and immunogenicity assessment of neoTSTs in liver cancer

**Figure 2:**
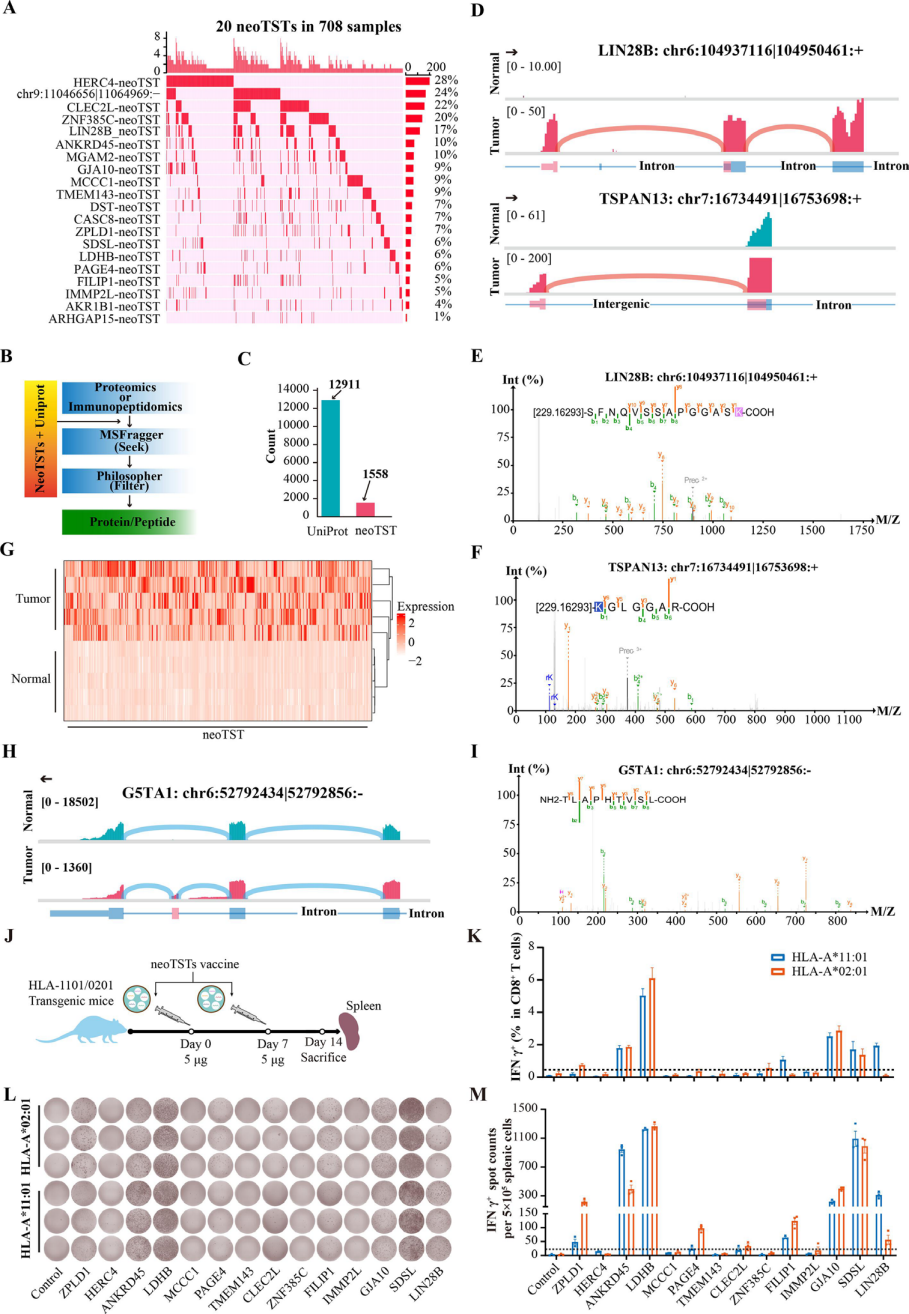
MS and immunopeptidomics-based identification and immunogenicity assessment of neoTSTs in liver cancer. (**A**) Distribution and expression frequency of 20 neoTSTs across samples. The waterfall plot illustrates the presence (red) or absence (white) of 20 neoTSTs (left panel) in the analyzed cohort. Each column represents an individual sample, and each row corresponds to a specific neoTST. Red indicates detectable expression of the neoantigen. (**B**) Overview of the proteomic and immunopeptidomic analysis pipeline. (**C**) The bar plot compares the total number of UniProt background proteins and neoTST-derived proteins detected across samples. Values represent the cumulative protein counts from all replicates/experimental conditions. (**D**) Sashimi plot of LIN28B-neoTST and TSPAN13-neoTST. (**E–F**) MS spectrum of peptides of LIN28B-neoTST and TSPAN13-neoTST. (**G**) The heatmap displays normalized expression levels of neoTST-derived peptides identified by MS. (**H**) MS spectrum of peptide of G5TA1-neoTST. (**I**) Sashimi plot of G5TA1-neoTST. (**J**) Experimental process of transgenic mice. (**K–M**) Flow cytometry quantification, ELISpot images and ELISpot quantification of IFN-γ-secreting CD8^+^T cells derived from two transgenic mice (HLA-A*02:01 and HLA-A*11:01) after stimulation with neoTST mRNA-LNP vaccine. The error bars represent the SE, while the horizontal dashed lines indicate the positive threshold. Areas above the dashed lines indicate that neoTSTs have induced specific T-cell responses. The data are presented as the mean±SE error of N independent cases (**K, M, **n=3). IFN-γ, interferon-gamma; LNP, Lipid Nanoparticles; mRNA, messenger RNA; MS, mass spectrometry; neoTSTs, neoantigen-encoding tumor-specific transcripts.

Compared with neoMuts (mean frequency <0.001), neoTSTs exhibited significantly higher population frequencies (mean frequency >0.02), suggesting their potential as shared neoantigens capable of broader patient coverage (figure 2A, online supplemental figure S2A). Independent analysis of the top 20 neoTST candidates demonstrated their broad patient applicability, directly targeting 708 cases. This demonstrates both the polyvalent nature of neoTSTs and the existence of shared epitopes across different neoTSTs, making the identification of identical neoTSTs in unmatched samples a feasible prospect. To validate the translational potential and population-wide sharedness of neoTSTs, we analyzed mass spectrometry data from 159 paired HCC and adjacent non-tumor samples (figure 2B). Peptide-level evidence was detected for 1,558 (3.9%) neoTSTs across the cohort, alongside 12,911 UniProt-annotated proteins (figure 2C, online supplemental figure S2B). Notably, specific neoTSTs such as LIN28B-neoTST and TSPAN13-neoTST were directly verified at the protein level (figure 2D–F). Mass spectrometry analysis confirmed the high tumor specificity of neoTSTs (figure 2G). 73.1% of neoTSTs provided ≥2 predicted epitopes matching patient-specific HLA haplotypes, with 59.4% capable of binding multiple HLA allelic subtypes (self-HLA), demonstrating superior immunogenic potential compared with neoMuts that typically yield single epitopes (online supplemental figure S2A). Further analysis of immunopeptidomic profiles from 15 patients with HCC revealed ≈3 neoTSTs presented HLA-I epitopes per patient after stringent filtering, with two-thirds originating from novel splice junctions (online supplemental figure S2C). Representative examples like G5TA1-neoTST (figure 2H,I) confirmed both the translational capacity and presentation superiority of neoTSTs. Analysis of HLA restriction patterns for immunopeptidome-validated neoantigens revealed significant allelic bias, with HLA-A*02:01 and HLA-B*07:02 emerging as dominant restriction elements (online supplemental figure S2D).

To validate endogenous immunogenicity, we selected 14 neoTSTs (online supplemental table S1) and conducted further experimental verification in HLA-A*02:01 and HLA-A*11:01 transgenic mice. Each candidate fragment contained at least one HLA-A*11:01-restricted or HLA-A*02:01-restricted epitope, with seven neoTSTs fragments harboring binding epitopes for both HLA subtypes. We designed and produced a mixed neoTST mRNA-LNP vaccine,^41^ which was administered via two i.m. injections (5 µg/dose) at a 7-day interval to humanized transgenic mice expressing either HLA-A*11:01 or HLA-A*02:01. 1 week after immunization, splenocytes were subjected to interferon-gamma (IFN-γ) ELISpot and flow cytometry to quantify antigen-specific T-cell responses (figure 2J). The flow cytometry results showed that 50% (7/14) of the neoTSTs elicited robust HLA-A*11:01-restricted or HLA-A*02:01-restricted T-cell activation, including LDHB, GJA10, ANKRD45, SDSL, ZPLD1, FILIP1 and LIN28B-neoTST (figure 2K). Moreover, 57.1% (4/7) of these activated T-cell responses were specific to both HLA subtypes transgenic mice. Notably, all flow-cytometry-positive neoTSTs were confirmed by ELISpot assay, which additionally included the T-cell response triggered by PAGE4-neoTST (figure 2L,M). The four double-positive neoTSTs exhibited particularly strong responses, with consistent patterns across mice, demonstrating the reliability of these findings and supporting neoTSTs as promising universal antigen candidates. Of note, among the 14 experimentally tested neoTSTs, only 4 were absolute neoTSTs and 10 were non-absolute neoTSTs, which is consistent with the overall proportion observed across our neoTST dataset (figure 1D). Importantly, even among non-absolute neoTSTs, candidates such as SDSL-neoTST exhibited potent immunogenicity, activating T cells in the context of both HLA-A*02:01 and HLA-A*11:01. These findings underscore the value of retaining non-absolute neoTSTs in our screening pipeline, as this category harbors promising immunogenic targets with high translational potential.

### Retention of non-coding sequences and transposon-driven activation generate high-yield neoantigen expansion

**Figure 3:**
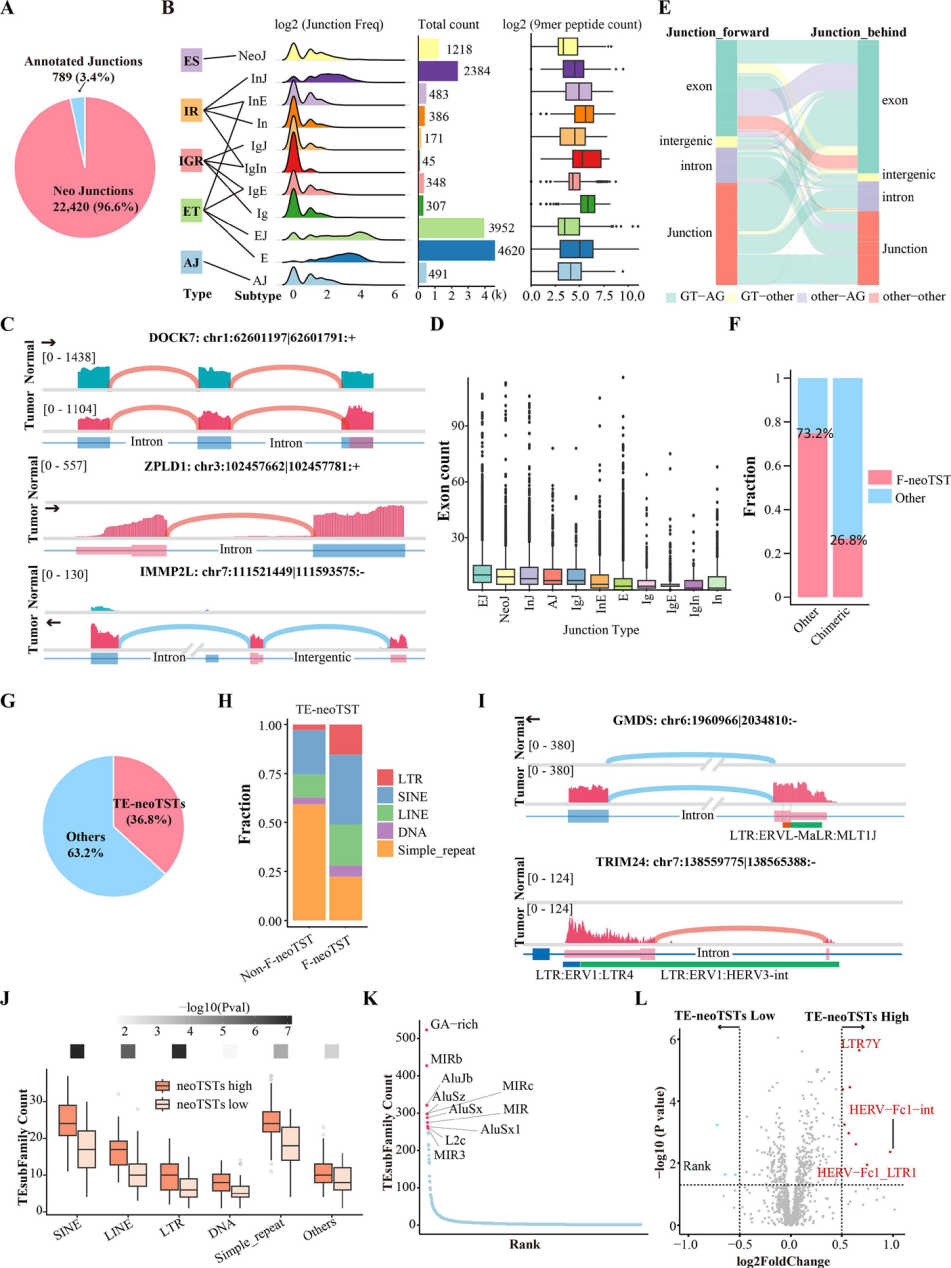
Retention of non-coding sequences and transposon-driven activation generate high-yield neoantigen expansion. (**A**) The pie chart illustrates the proportion of annotated (pink) versus unannotated (light blue) TSJs identified in liver cancer. (**B**) Characterization of five major tumor-specific splicing types and their 11 reference genome location sources. Left panel: Density plot showing the distribution frequency. Middle panel: Bar plot displaying the total count of TSJ events. Right panel: Boxplot comparing the number of 9-mer peptides from neoTSTs for each TSJ type. The specific classifications can be found in the online supplemental material. (**C**) Sashimi plots of DOCK7 (ET), ZPLD1 (IR), and IMMP2L (IGR). (**D**) The boxplot compares the number of exons contained within neoTSTs generated by distinct splicing event subtypes. (**E**) The Sankey diagram illustrates the distribution of splicing signals (colored links) derived from different reference genome locations (left nodes). Non-canonical splicing signals (colored in non-green hues) are contrasted with canonical GT-AG signals (green). Transparent link opacity emphasizes signal flow density, with node widths proportional to the frequency of genomic region contributions. (**F**) Bar plot compares the percentage of F-neoTSTs within PNI-neoTSTs (left bar) and FNT-neoTSTs (right bar). PNI: including the exons of some annotated transcripts. FNT-neoTSTs: completely composed of brand-new exons. (**G**) The proportions of TE-neoTSTs and non-TE-neoTSTs. (**H**) The stacked bar plot compares the distribution of TE subfamilies between F-neoTSTs (functional, left bars) and non-F-neoTSTs (non-functional, right bars) derived from TE-associated splicing events (TE-neoTSTs). (**I**) Sashimi plot of 2 TE-neoTSTs: GMDS-neoTST and TRIM24-neoTST. (**J**) The boxplot compares the expression levels of individual TE classes between high neoTSTs burden samples and low neoTSTs burden samples. Each box represents the distribution of subfamily expression within a TE type. Dots indicate outliers. (**K**) Number of neoTSTs derived from individual TE subfamilies, ordered by descending count (left to right on the x-axis). The y-axis represents the absolute count of neoTSTs per subfamily. (**L**) The volcano plot displays log2 fold-change (x-axis) and statistical significance (−log10 adjusted p value, y-axis) of TE subfamily expression in high versus low TE-neoTST burden groups. ES, exon skip; ET, exon truncation; FNT, fully novel transcript; IGR, intergenic region retention; IR, intron retention; neoTST, neoantigen-encoding tumor-specific transcript; PNI, partially novel isoform; TE, transposable element; TSJs, tumor-specific junctions; TSS, transcriptional start site.

Genomic annotation revealed that 96.6% of neoTSTs originate from unannotated splicing sites (figure 3A), highlighting the critical role of alternative mechanisms in neoantigen generation. To systematically characterize splicing diversity, we classified aberrant splicing events into four categories: exon skip (ES), exon truncation (ET), intron retention (IR), and intergenic region retention (IGR) (online supplemental figure S3A). Quantitative analysis demonstrated that neoTSTs predominantly arise from ET events (68%), followed by IR (18%), IGR (6%) and ES (6%), with only a minor contribution from IGR (2%) (online supplemental figure S3B). Representative examples of ET (eg, DOCK7-neoTST), and IR (eg, ZPLD1-neoTST) and IGR (eg, IMMP2L-neoTST) events are illustrated in figure 3C. Notably, IR and IGR events exhibited superior neoantigen-generating capacity, producing 2.3-fold more presentable peptides compared with single-site anomalies. Multisite aberrant events (both before and after splicing are unannotated splicing sites) further enhanced neoantigen yield (figure 3B), attributable to their truncated ORFs (figure 3D). These shortened ORFs confer dual advantages: (1) accelerated ribosomal initiation and elongation,^42^ and (2) enhanced peptide processing and TAP-dependent transport,^43^ collectively optimizing neoantigen presentation. While most splicing junctions adhered to canonical GT-AG signals (online supplemental figure S3C), exon-exon splicing exhibited widespread non-canonical signal selection (eg, GC-AG, AT-AC; figure 3E). These findings establish that beyond coding-region frameshifts (ET/ES), non-coding sequence retention (IR/IGR) serves as a potent neoantigen source, with their truncated ORFs potentially enhancing immunogenicity through streamlined biogenesis and presentation pathways.

Alternative promoter usage through transcription start site (TSS) selection represents a significant source of neoTST generation.^44^ Our analysis revealed that 67.9% of neoTSTs consisted entirely of novel exons (fully novel transcript neoTSTs), while 32.1% were chimeric transcripts (partially novel isoform neoTSTs: PNI-neoTSTs) (online supplemental figure S3D,E). Notably, 26.8% of PNI-neoTSTs used novel TSS loci (F-neoTSTs), suggesting their regulation by alternative promoters (figure 3F). Amino acid position analysis of neo-peptides (neoPeps) demonstrated strong enrichment at N-terminal chimeric regions (online supplemental figure S3F), further supporting alternative promoter activity as a key driver of neoTST biogenesis. Given the established role of TEs in generating aberrant splicing isoforms^26^ and their documented promoter activity in cancer,^45 46^ we systematically analyzed TE content within the first exon and 200 bp upstream region of neoTSTs (TE-neoTSTs). Strikingly, 36.8% of neoTSTs harbored TE sequences in these regulatory regions (figure 3G). Among these TE-neoTSTs, F-neoTSTs exhibited significant enrichment of LTR (~sixfold), SINE (~1.5-fold), and LINE (~twofold) elements compared with non-F-neoTSTs, with LTR elements showing particularly strong association (figure 3H,I). These findings align with prior reports of LTR-specific promoter activity^47 48^ and collectively demonstrate that TEs—especially LTR, SINE, and LINE elements—may function as alternative promoters to activate neoTSTs transcription in cancer.

To substantiate whether TEs drive neoTSTs expression, we quantified TE expression levels across all patients with HCC. Analysis revealed significantly elevated TE expression in patients with high neoTSTs burden (p<0.001, figure 3J). Characterization of TE-associated neoTSTs subtypes identified distinct TE subfamily enrichments: GA-rich elements predominated in non-F-neoTSTs, while Alu and MIR elements were enriched in F-neoTSTs (figure 3K, online supplemental figure S3G). Differential expression analysis further demonstrated that upregulated TEs clustered predominantly in high-neoTST-burden samples (figure 3L). Notably, LTR7Y expression exhibited a proportional correlation with neoTST burden (p=1.2e-7, online supplemental figure S3H). These findings collectively demonstrate that TE-specific activation may provide alternative promoters that potentiate neoTSTs expression in HCC. In addition to regulating the transcription of neoTSTs, amino acid sequences derived from TEs can also serve as a substantial source of neoPeps. We characterized TE-chimeric neoTSTs (Methods) and found that 21.4% of neoTSTs were TE-neoTSTs, generating NeoPeptides derived from TE sequences (online supplemental figure S3I).

### NeoTSTs could generate de novo transmembrane domains

**Figure 4:**
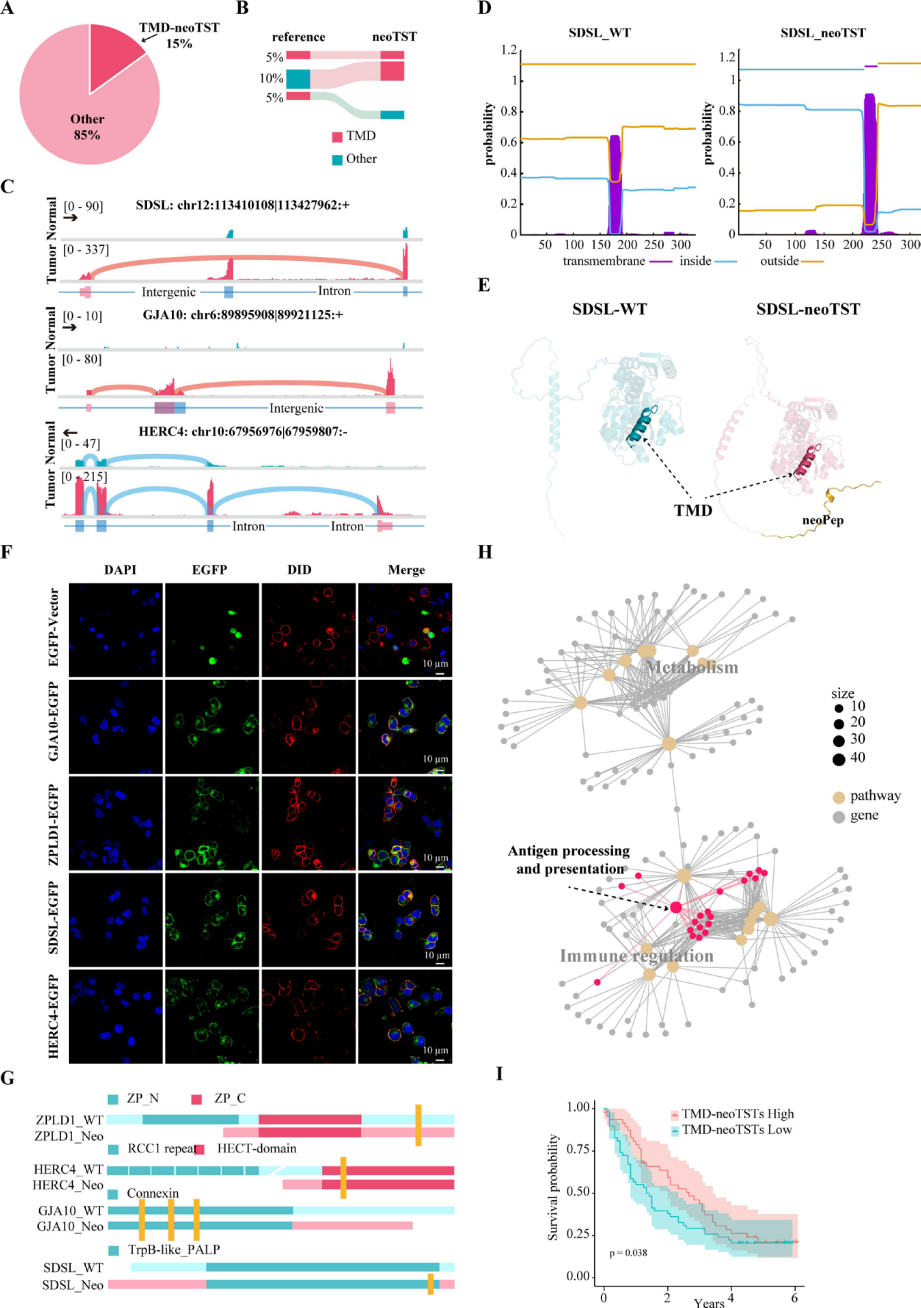
neoTSTs could generate de novo TMDs. (**A**) The proportions of TMD-neoTSTs. (**B**) TMD dynamics between reference proteins and neoTST-derived isoforms. The Sankey diagram visualizes changes in TMD status (presence/absence) between reference protein isoforms (left nodes) and neoTST-derived isoforms (right nodes). Node colors: Red=TMD present, Green=TMD absent. Link colors: TMD gained or retained in neoTSTs, Green=TMD lost in neoTSTs. Link widths correspond to the frequency of each transition. (**C**) Sashimi plot of three TMD-neoTSTs: SDSL-neoTST, GJA10-neoTST and HERC4-neoTST. (**D**) TMD prediction profiles along the protein sequence (x-axis: residue position; y-axis: probability). Purple: predicted transmembrane domains (probability ≥0.7); blue line: cytoplasmic regions; yellow line: extracellular regions. (**E**) The figure presents the three-dimensional structural models of: WT SDSL (blue ribbon diagram) SDSL-neoTST (red ribbon diagram). (**F**) The panel displays immunofluorescence images of four TMD-neoTSTs (rows), each visualized with four markers (columns): DAPI (blue): nuclear staining. GFP (green): TMD-neoTST fusion protein expression. DID (red): membrane dye (labels plasma membrane or organelles). Merge: overlay of all channels. (**G**) Domain annotation of four TMD-neoTSTs and their wild-type proteins. Yellow vertical bars: TMDs. (**H**) The network diagram illustrates functional associations between wild-type proteins (gray nodes) and their enriched biological pathways (yellow nodes), with antigen processing and presentation pathways highlighted in red. (**I**) Kaplan-Meier survival curves for the in-house cohort stratified by TMD-neoTST burden. The mean TMD-neoTST burden was used for stratification. DAPI, 4',6-diamidino-2-phenylindole; neoTST, neoantigen-encoding tumor-specific transcript; TMD, transmembrane domain; WT, wild-type.

The generation of immunogenic neoantigens requires proteolytic processing and TAP-mediated transport of peptides into the endoplasmic reticulum for major histocompatibility complex (MHC)-I loading, a process potentially facilitated by TMDs. Our analysis revealed that approximately 15% of neoTSTs contained TMDs, with 67% representing novel TMDs acquired through chimeric transcript formation (figure 4A,B). While wild-type membrane proteins like ZPLD1-neoTST, HERC4-neoTST and GJA10-neoTST retained their original TMDs after alternative splicing (figure 4C), non-membrane proteins such as SDSL-neoTST gained de novo transmembrane capability through structural reorganization (figure 4D). Strikingly, despite significant alternative splicing-induced modifications, all four TMD-containing neoTSTs maintained functional transmembrane topology (figure 4E, online supplemental figure S4A). Immunofluorescence validation confirmed membrane localization not only for TMD-neoTSTs derived from native membrane proteins (ZPLD1-neoTST, HERC4-neoTST, GJA10-neoTSTs) but also for SDSL-neoTST, demonstrating the widespread acquisition of membrane-targeting capacity among neoTSTs (figure 4F). In vivo validation showed three of four tested membrane-capable neoTSTs (including the de novo transmembrane SDSL-neoTST and multi-TMD GJA10-neoTST) potently activated HLA-restricted T cells (figure 2I–K).

Functional domain annotation revealed that TMD-neoTST-derived membrane domains frequently occupied critical protein regions (figure 4G), suggesting preserved functional capacity. Notably, most membrane-capable neoTSTs originated from originally non-transmembrane proteins (figure 4B). Enrichment analysis demonstrated significant association with antigen processing/presentation pathways alongside metabolic functions (figure 4H). Clinically, patients with higher TMD-neoTST burden exhibited improved survival (figure 4I), suggesting that acquired transmembrane functionality may enhance neoantigen presentation efficiency and ultimately improve patient outcomes through structural and functional modulation of antigen processing.

### HNF4A binding activates neoTSTs transcription in HCC

**Figure 5:**
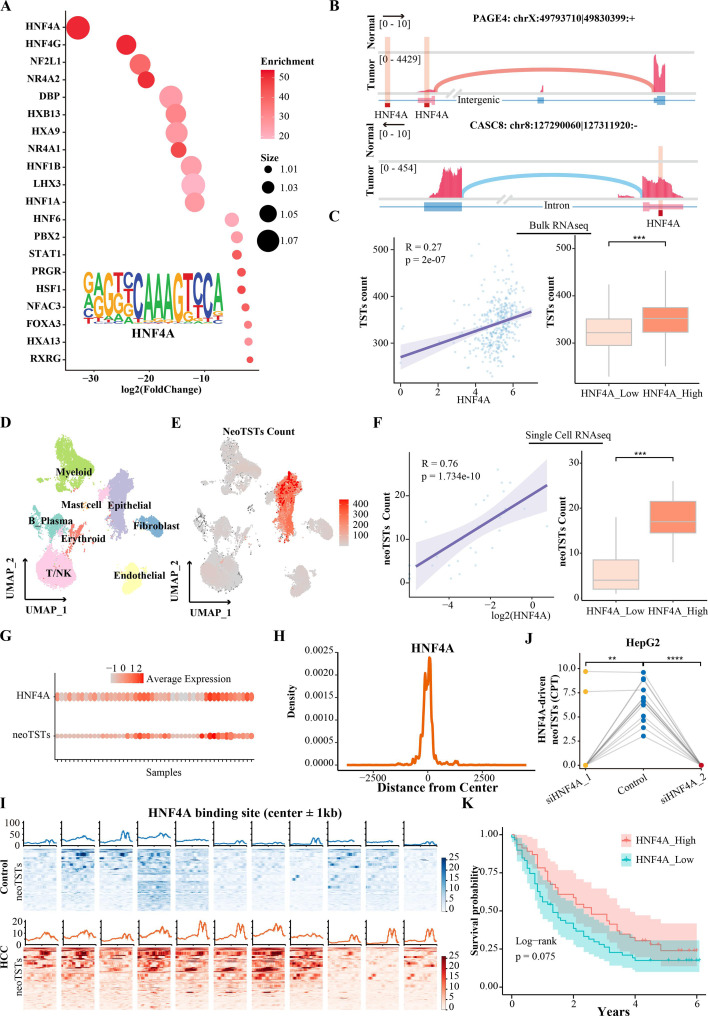
HNF4A binding activates neoTSTs transcription in HCC. (**A**) The bubble plot illustrates TF enrichment in the transcriptional start regions of F-neoTSTs. (**B**) Sashimi plot of two HNF4A binding neoTSTs: PAGE4-neoTST and CASC8-neoTST. (**C**) The panel combines two analyses of bulk RNA-seq data to link HNF4A expression with neoTST load. (**D**) The UMAP plot displays clustering of single cells (dots) from liver cancer tissues. (**E**) The UMAP plot highlights neoTSTs count within the single-cell landscape of liver cancer. (**F**) The panel combines two analyses of single-cell RNA-seq data to link HNF4A expression with neoTST load. (**G**) The dot plot displays the relationship between HNF4A expression and neoTST burden at the single-cell RNA-seq data across patient samples. Rows: Individual patient samples. (**H**) The schematic illustrates putative HNF4A binding motifs within a 6 kb window (x-axis: position relative to the TSS of F-neoTSTs; range: −3 kb to +3 kb from TSS, marked by vertical dashed line). (**I**) ATAC-seq signals (±1 kb around HNF4A binding sites) between 11 HCC tumors (red) and 11 normal liver tissues (blue), aligned to the center of HNF4A peaks (x-axis: genomic position; y-axis: normalized ATAC-seq signal intensity). (**J**) HNF4A-driven neoTST expression (CPT) in HepG2 between HNF4A knockdown group (siHNF4A_1, siHNF4A_2) and non-knockdown group (Control). (**K**) Kaplan-Meier survival curves for the in-house cohort stratified by HNF4A expression. The mean FPKM of HNF4A was used for stratification. ATAC, Assay for Transposase-Accessible Chromatin; FPKM, Fragments Per Kilobase of transcript per Million mapped reads; CPT, coverage per 10 million reads; HCC, hepatocellular carcinoma; neoTST, neoantigen-encoding tumor-specific transcript; NK, natural killer; RNAseq, RNA sequencing; TF, transcription factor; TSS, transcriptional start site; TSTs, tumor-specific transcripts; UMAP, uniform manifold approximation and projection. Statistical significance was determined by Wilcoxon rank-sum test (**p < 0.01, ***p < 0.001, ****p < 0.0001).

To further explore potential regulatory mechanisms of neoTSTs, we performed transcription factor enrichment analysis on the first exon regions of F-neoTSTs. Our results revealed significant enrichment of transcription factors such as HNF4A and HNF4G in the initiation regions of F-neoTSTs. Notably, the computational prediction and correlation analysis suggest that HNF4A may have a potential binding and regulatory relationship with a considerable portion (51.03%) of the promoter region of F-neoTST, and its binding level showed a significant positive correlation with neoTST burden (figure 5A). For instance, both PAGE4 and CASC8-neoTSTs contained HNF4A-binding sites within their promoter regions (figure 5B). Furthermore, analysis of bulk RNA-seq data confirmed that HNF4A expression was positively correlated with neoTST burden, with HNF4A-high patients exhibiting a greater number of neoTSTs (figure 5C). Single-cell RNA-seq data with 140 HCC samples across 20 datasets^49^ were integrated to further elucidate the regulatory mechanism of HNF4A on neoTSTs. We quantified neoTSTs in the tumor cells of each patient. Some previously detected neoTSTs were selected for observation, such as LIN28B-neoTST and DST-neoTST. It can be seen that these neoTSTs are highly enriched in tumor cells and are hardly expressed in other cells. This further corroborates the credibility of our quantitative results (online supplemental figure S5A–C). We observed that the expression of HNF4A was highly specific to liver cancer cells and showed significant colocalization with neoTSTs expression (figure 5D–F, online supplemental figure S5D). Statistical analysis further confirmed a strong positive correlation between HNF4A expression and neoTSTs burden at the single-cell level (figure 5G), supporting a functional link between them.

The analysis revealed a significant central enrichment phenomenon at the binding site of HNF4A in the promoter region of F-neoTSTs (figure 5H). To investigate whether HNF4A regulates neoTSTs coupling with epigenetic mechanisms, we analyzed Assay for Transposase-Accessible Chromatin with sequencing (ATAC-seq) data from 22 internal samples (11 controls and 11 HCC cases). The results revealed that potential HNF4A binding sites exhibited significantly increased chromatin accessibility in patients with HCC, with these sites predominantly located in non-coding regions such as promoters and enhancers. This suggests that tumor-specific epigenetic opening may recruit HNF4A binding, thereby activating neoTSTs transcription (figure 5I). To further validate HNF4A’s regulation of neoTST biogenesis, we analyzed RNA-seq data from HepG2 cells following HNF4A knockdown (GSE199068, two independent siRNA constructs). Both knockdown conditions showed significant reductions in HNF4A-driven neoTSTs (figure 5J), establishing a causal relationship between HNF4A expression and neoTST generation. Survival analysis further indicated that high HNF4A expression was associated with favorable patient prognosis (figure 5K).

### Intercellular distribution heterogeneity of neoTSTs at the single-cell resolution

**Figure 6:**
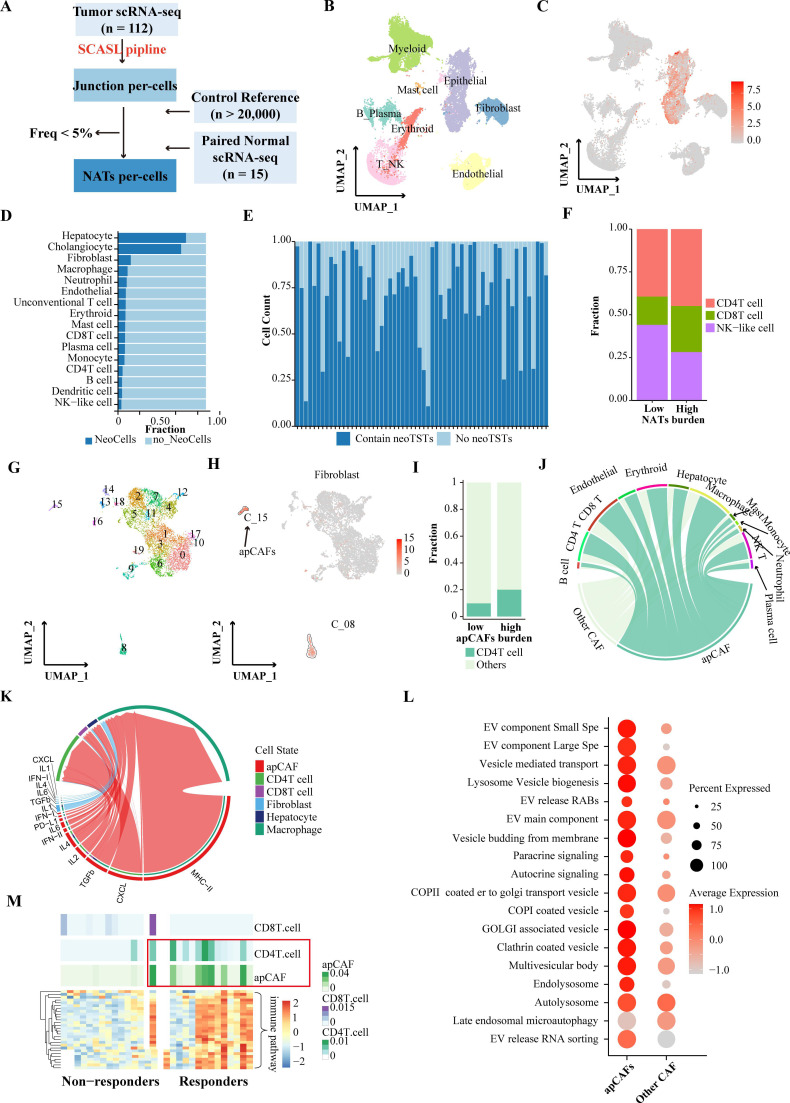
Heterogeneity of neoTSTs at single-cell resolution. (**A**) Workflow for single-cell level identification of neoTSTs. (**B**) The UMAP plot displays clustering of single cells (dots) from HRA001748. (**C**) UMAP plot displays the landscape of neoTST count across individual cells in the HCC. (**D**) Stacked bar plot quantitatively compares the prevalence of neoTSTs among different cell populations in HCC. (**E**) Stacked bar plot quantitatively compares the prevalence of neoTSTs among different samples in HCC. (**F**) Stacked bar plot quantitatively compares the prevalence of T cells between high and low neoTST burden samples. (**G**) The UMAP plot shows the result of all the re-clustering of CAFs. (**H**) UMAP plot displays the landscape of neoTST count across individual cells in all CAFs. (**I**) Stacked bar plot quantitatively compares the prevalence of CD4^+^ T cells between high and low apCAFs burden samples. (**J**) Chord diagram, generated through CellChat analysis, maps the signaling crosstalk between apCAFs/other CAFs, and major cell populations in HCC. (**K**) Chord diagram highlights the immune-modulatory interactions initiated by apCAFs and other CAFs. (**L**) Dotplot compares the exosome-associated signaling pathway enrichment scores between apCAFs and other CAF subtypes in HCC. (**M**) Heatmap visualization presents a comprehensive comparison of immune microenvironment features between R and NR patient groups. Top panel: four cellular features (CD4^+^ T cells, CD8^+^ T cells, apCAFs, other CAFs). Bottom panel: 15 key immune pathways (grouped by function). Color gradient: Blue (low), white (medium), red (high) normalized pathway activity. apCAFs, antigen-presenting cancer-associated fibroblasts; CAF, cancer-associated fibroblast; EV, extracellular vesicle; HCC, hepatocellular carcinoma; IFN, interferon; IL, interleukin; MHC, major histocompatibility complex; neoTST, neoantigen-encoding tumor-specific transcript; NK, natural killer; NR, non-responsive; R, treatment-responsive; scRNA-seq, single-cell RNA sequencing; UMAP, uniform manifold approximation and projection.

HCC exhibits extensive tumor heterogeneity and a complex immune microenvironment, necessitating analysis of neoTST heterogeneity at single-cell resolution. We collected single-cell RNA-seq data from a cohort of 112 patients with liver cancer and 15 healthy donors, and developed a computational pipeline for quantifying and screening neoTSTs at the single-cell level (figure 6A). After reclustering, we annotated patient cells into 16 distinct cell types, including tumor cells (HCC and ICC tumor cells), 12 immune cell subtypes, and 2 stromal cell types (figure 6B). We observed that neoTSTs are primarily produced by tumor cells, enriched in tumor epithelial cells (figure 6C). Although the proportion of cancer cells expressing neoTSTs varied among patients, and the specific neoTSTs expressed differed across cancer cells, neoTSTs collectively covered over 75% of HCC cells in the cohort (figure 6D). These findings suggest that personalized vaccine design should target multiple neoTST antigens to achieve optimal therapeutic efficacy. To further investigate cellular heterogeneity, we stratified patients into low-neoTST and high-neoTST burden groups based on the proportion of tumor cells expressing neoTSTs (figure 6E). Analysis of T cell composition showed that the proportion of CD8^+^ T cells in the high-burden group was more than double that in the low-burden group, indicating a heightened state of immune activation in high-neoTST tumors (figure 6F). These results further support the role of neoTSTs in promoting immune activation in patients with HCC.

In addition to hepatocarcinoma cells, neoTSTs were also detected in stromal and immune cells of some patients, with fibroblasts showing the highest abundance (figure 6D). We extracted cancer-associated fibroblasts (CAFs) from all patients with HCC and performed reclustering analysis. CAFs were grouped into 19 clusters, with neoTSTs predominantly enriched in clusters C_08 and C_15 (figure 6G,H, online supplemental figure S6A). Previous studies have identified a subset of apCAFs that express MHC-II molecules and present MHC-II antigens.^50^ Further annotation revealed that C_15 and C_08 corresponded to apCAFs and myofibroblastic CAFs, respectively (online supplemental figure S6B). Patient stratification based on apCAF proportion showed that patients with high apCAF levels had significantly increased CD4^+^ T cell infiltration, consistent with MHC-II expression by apCAFs (figure 6I). Moreover, cell–cell interaction analysis indicated that apCAFs had significantly stronger interactions with CD4^+^ T cells compared with other fibroblasts, primarily involving MHC-II-related pathways (figure 6J,K, online supplemental figure S6C). These findings suggest that apCAFs may activate CD4^+^ T cells and promote MHC-II-restricted antigen presentation. Further analysis shows the majority of extracellular vesicles-related pathway components upregulated in apCAFs relative to other CAFs (figure 6L). By analyzing the microenvironmental characteristics of 27 patients with HCC undergoing immunotherapy (14 responded and 13 did not respond) using CIBERSORTx, it was found that the enrichment level of apCAFs in the responding group was significantly positively correlated with the density of CD4^+^ T cells (figure 6M). These results highlight the potential significance of fibroblasts in immunotherapy.

### NeoTST elicited antigen-specific CD8^+^ and CD4^+^ T cell responses and inhibited tumor growth in a syngeneic HCC model

**Figure 7:**
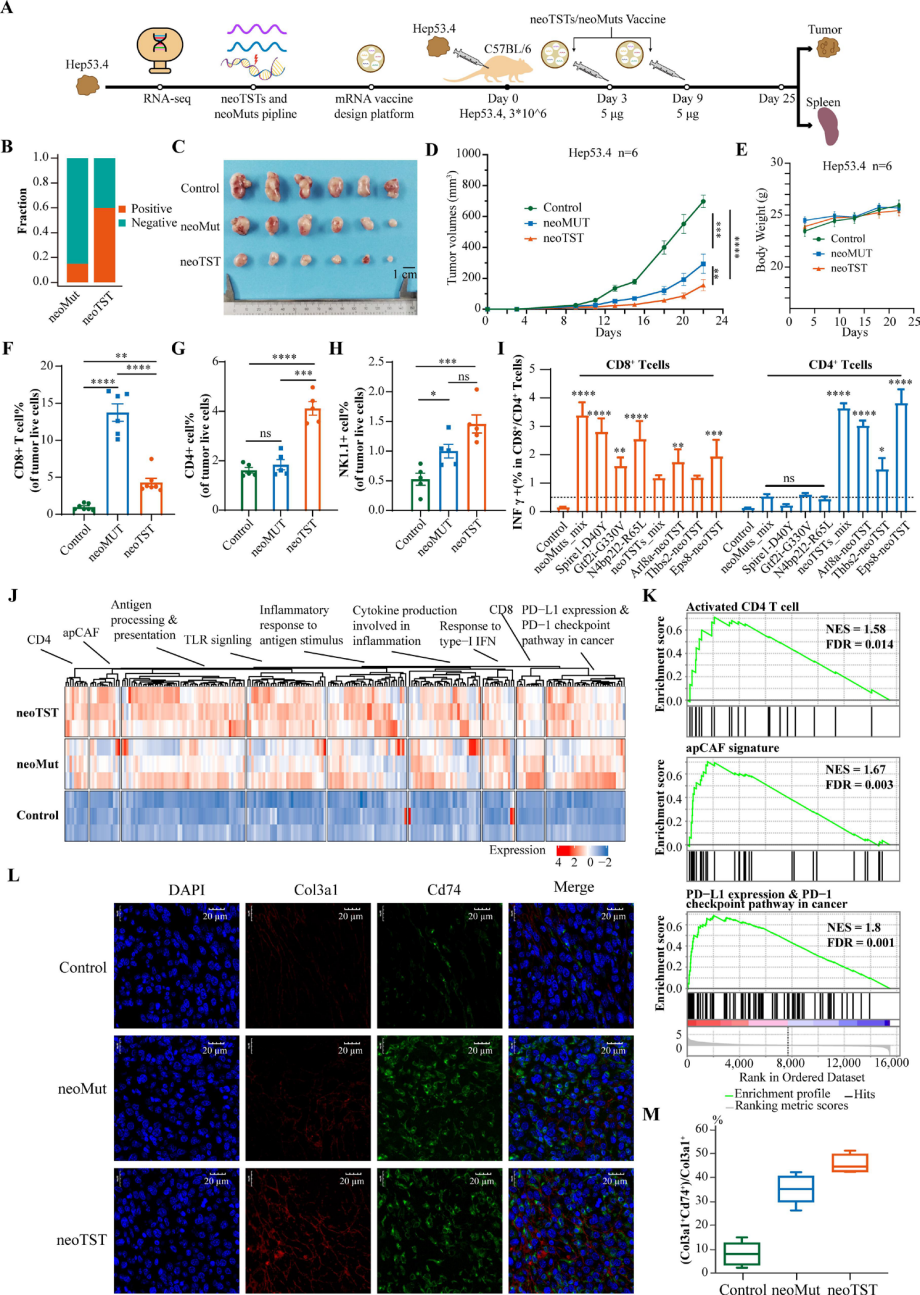
neoTST elicited antigen-specific CD8^+^ and CD4^+^ T cell responses and inhibited tumor growth in a syngeneic HCC model. (**A**) Schematic of using the murine hepatocellular carcinoma cell line Hep53.4 as a model system to validate in vivo immunogenicity of neoTSTs and assess their therapeutic potential. (**B**) Stacked bar plot of neoMuts and neoTSTs positivity rates by flow cytometry. (**C**) Tumor size measurement and visualization. (**D**) Tumor volume progression (mean±SEM) over time in different mouse treatment cohorts. (**E**) Body weight progression (mean±SEM) over time in different mouse treatment cohorts. (**F–H**) Cell fraction (mean±SEM) of CD8^+^ T cells, CD4^+^ T cells, and NK T cells in different mouse treatment cohorts. (**I**) Proportions of CD8^+^ T cells (left) and CD4^+^ T cells (right) induced by neoTST versus neoMut neoantigens. Control: unvaccinated mice. (**J**) Clustered heatmap compares the immune microenvironment profiles of different treatment groups in the mouse model, integrating immunity pathway and cell-type-specific gene expression. (**K**) GSEA results comparing neoTST-treated versus control mice. (**L**) Representative IF staining images of Col3a1 (red) and Cd74 (green) in the tumors from indicated groups. (**M**) Fraction of apCAF in CAFs ((Col3a1^+^Cd74^+^)/Col3a1^+^). apCAF, antigen-presenting cancer-associated fibroblast; CAFs, cancer-associated fibroblasts; DAPI, 4',6-diamidino-2-phenylindole; FDR, False Discovery Rate; GSEA, gene set enrichment analysis; HCC, hepatocellular carcinoma; IF, immunofluorescence; IFN-γ, interferon-gamma; mRNA, messenger RNA; neoMuts, mutation-derived neoantigens; neoTSTs, neoantigen-encoding tumor-specific transcripts; NES, Normalized Enrichment Score; NK, natural killer; ns, not significant; PD-1, programmed cell death protein-1; PD-L1, programmed death-ligand 1; RNA-seq, RNA sequencing; TLR, Toll-like Receptor. One-way ANOVA was used; data shown as mean±SEM. *P<0.05, **P<0.01, ***P<0.001, ****P<0.0001.

To assess the immunogenicity and therapeutic efficacy of neoTST-derived neoantigen vaccines in vivo, we established a murine HCC model using the Hep53.4 cell line (figure 7A). Through comprehensive genomic profiling, we identified five neoTSTs and 20 neoMuts in this system (figure 7B, online supplemental figure S7A,B, online supplemental tables S2–4). The neoTST identification pipeline mirrored our human analysis protocol, comprising four key steps: (1) transcriptome assembly, (2) reference database construction, (3) TST detection, and (4) neoantigen prediction and validation. For neoMut identification, we detected non-synonymous single nucleotide variants (SNVs) and cross-referenced these against murine genomic databases. Candidate neoMuts were subsequently prioritized using MHCPan prediction algorithms, yielding 20 high-confidence targets (online supplemental table S5). We designed and produced a mixed mRNA-LNP vaccine (a vaccine composed of multiple mRNAs), which was administered via two i.m. injections (5 µg/dose) at a 7 day interval to C57BL/6 mice. Flow cytometry analysis demonstrated the superior immunogenicity of neoTSTs, with 60% (3/5, Arl8a, Thebs2, Eps8-neoTSTs) eliciting strong concurrent CD4^+^ and CD8^+^ T-cell responses compared with only 15% (3/20, Spire1, Gtf2i N4bp212-neoMuts) of neoMuts eliciting CD8^+^ T-cell responses (figure 7B, online supplemental figure S7C). Notably, the three immunogenic neoTSTs originated from IR events, highlighting non-coding region retention as a critical neoantigen source, and one strongly responsive target (Eps8-neoTST) was driven by a SINE element.

Furthermore, mice bearing subcutaneous Hep53.4 tumors were vaccinated with the respective neoantigen vaccines on days 5 and 12 post-tumor inoculation. Tumor growth was monitored, and after 21 days, subcutaneous tumors and spleens were harvested. Results showed that both mutation-derived and TST-derived neoantigens effectively suppressed tumor growth, with neoTSTs demonstrating superior efficacy (figure 7C,D). Body weight monitoring indicated no significant systemic toxicity, suggesting favorable safety profiles of the vaccines (figure 7E). Comparative analysis of TILs revealed distinct immune activation patterns between vaccination groups. Mice immunized with neoMuts exhibited significantly higher CD8^+^ T cell infiltration compared with both control (p<0.001) and neoTSTs (p<0.01) groups (figure 7F). Notably, while neoMuts vaccination showed no significant effect on CD4^+^ T cell recruitment, neoTSTs immunization induced robust CD4^+^ T cell infiltration (p<0.001 vs control), demonstrating their unique capacity to activate both MHC class I and II antigen presentation pathways (figure 7G). Both vaccine groups showed increased natural killer cell infiltration relative to controls (p<0.05), though the difference between neoMuts and neoTSTs groups did not reach statistical significance (figure 7H). These results indicate that neoTSTs can simultaneously activate the immune pathways of both MHC I and MHC II, and they exhibit a stronger cytotoxic potential compared with neoMuts. Following independent transfection of the three neoMuts and three neoTSTs, flow cytometric analysis revealed distinct immunogenic profiles. While both vaccine types elicited antigen-specific CD8^+^ T cell responses, only neoTSTs demonstrated the capacity to induce robust CD4^+^ T cell activation (figure 7I). These findings provide direct experimental evidence that neoTSTs uniquely engage both MHC class I and II antigen presentation pathways, establishing their superior therapeutic potential compared with neoMuts for comprehensive antitumor immunity.

### neoTST enhances the infiltration of apCAF in a syngeneic HCC model

To further compare the effects of neoantigen vaccines, we collected Hep53.4 tumor tissues from mice that were either unvaccinated or vaccinated with neoMuts-based or neoTSTs-based vaccines for RNA-seq. Transcriptomic profiling revealed that both neoantigen vaccines robustly activated hallmark immune pathways, including antigen processing and presentation, cytokine production involved in inflammation, programmed death-ligand 1 (PD-L1) expression and PD-1 checkpoint pathway in cancer, inflammatory response to antigen stimulus, response to type-I IFN, Toll-like Receptor (TLR) signaling (figure 7J). Additionally, both vaccines potently upregulated gene signatures associated with activated CD4^+^ T cells, activated CD8^+^ T cells, and apCAF, with the neoTST vaccine demonstrating stronger activation than the neoMut vaccine. GSEA analysis further revealed distinct response patterns: while neoTST-vaccinated mice showed significant enrichment of Activated CD4^+^ T cell (Normalized Enrichment Score (NES)=1.58, False Discovery Rate (FDR)=0.014), apCAF signatures (NES=1.67, FDR=0.003), and PD-L1 expression and PD-1 checkpoint pathway compared with controls (NES=1.8, FDR=0.001), only PD-L1 expression and PD-1 checkpoint pathway was markedly activated in neoMut-treated mice (NES=1.70, FDR=0.01) (figure 7K, online supplemental tables S6 and S7). This also supported that neoTSTs may promote the CD4^+^ T cell immune pathway through apCAFs. Quantitative immunofluorescence revealed significantly enhanced apCAF (Col3a1^+^Cd74^+^) infiltration in neoTST-vaccinated tumors compared with control, whereas neoMut-vaccinated tumors showed intermediate infiltration levels (figure 7L,M), confirming the unique capacity of neoTST vaccines to remodel the tumor stroma through apCAF mobilization. These findings demonstrate that neoTSTs may exhibit superior antigen-presenting capacity compared with neoMuts. Furthermore, neoantigen vaccine stimulation robustly activates diverse immune pathways, driving the conversion of immunologically “cold” tumors into “hot” ones. This transformation creates a permissive microenvironment for enhanced efficacy when combined with ICIs, offering a promising combinatorial immunotherapy strategy.

## Discussion

Our study systematically identified an average of 60 neoTSTs per patient across 1,013 liver cancers through a stringent analytical pipeline. These neoTSTs predominantly originated from aberrant splicing events and IR, driven by TEs and the transcription factor HNF4A. Interestingly, besides tumor cells, apCAFs were also found to produce a subset of neoTSTs, which primarily encode MHC-II-restricted epitopes and facilitate CD4^+^ T cell activation. Functional validation in an HLA-A transgenic model confirmed the ability of neoTSTs to elicit specific T cell responses, and further experiments in HCC mouse models demonstrated their superior capacity in activating antitumor immunity and suppressing tumor growth. Collectively, our work identifies a large repertoire of therapeutically promising neoTSTs in HCC, offering novel targets and insights to advance immunotherapy and therapeutic development for liver cancer.

In recent years, growing attention has been drawn to the importance of transcript-derived neoantigens in patients with cancer. Previous studies have shown that analyzing alternative splicing enables more accurate identification of transcript isoforms.^27^ Conventional approaches for assessing transcript splicing, such as Percent Spliced In analysis, primarily quantify splicing events but are unable to directly identify novel splicing junctions and rely heavily on existing annotations. Recently, several tools-such as SPLICE-neo,^51^ SNAF,^52^ and IRIS^53^—have been developed to screen immunotherapeutic targets by detecting specific splicing sites. However, these tools predominantly focus on spliced events and often overlook transcripts without splicing. Moreover, many studies rely solely on splicing references from resources like GTEx Portal, which may limit the ability to comprehensively capture TSTs and introduce false positives. Additionally, we introduce a novel workflow that integrates RNA-seq data from diverse tissues, populations, and platforms to construct a comprehensive reference dataset. Through multilevel comparisons, our approach identifies both multiexon and single-exon neoTSTs, thereby expanding the repertoire of detectable neoantigens and improving prediction accuracy. Previous research suggests that shorter ORFs facilitate neoantigen processing and presentation, indicating that single-exon neoTSTs may possess stronger immunogenic potential.^42 43^ We also designed a unique alignment strategy: a hash-formatted reference peptide pool was built from known protein sequences, followed by prealignment of full-length neoTST protein sequences to identify novel specific peptides before further neoantigen prediction. This method reduces false positives while retaining a broad spectrum of potential neoantigens and significantly accelerates computational processing. Furthermore, unlike conventional methods that apply differential expression thresholds (eg, IRIS) or global fold-change filters (eg, SNAF), we established a comprehensive reference dataset encompassing three biological categories: 30 normal tissue types, matched adjacent non-tumor tissues, and non-cancer disease specimens. Each layer underwent independent benchmarking before feature integration. This multitiered comparison enables precise identification of tumor-specific splicing events, avoiding signal dilution caused by mixed tissue types or physiological states, thereby substantially enhancing neoantigen specificity. Notably, during the finalization of our study, a contemporaneous study identified transcript-derived neoantigens in HCC through alternative splicing analysis^54^; however, this work was limited to comparisons with only adjacent normal liver tissues, exhibited a pronounced false-positive rate, and crucially, did not address the epigenetic-driven transcriptional variants identified in our work. Overall, our novel workflow improves the accuracy of transcript-derived neoantigen identification and expands the candidate target pool for liver cancer.

In contrast to the highly individualized neoantigens derived from SNVs, neoTSTs originate from distinct molecular mechanisms-namely, aberrant splicing events and chromatin accessibility alterations. This unique origin confers two key advantages to neoTSTs. First, their splicing patterns exhibit reproducibility, enabling the possibility of shared neoTSTs across different patients with liver cancer (ie, “public neoantigens”). This characteristic opens avenues for developing universal T-cell therapies or vaccines, thereby overcoming the limitations of personalized mutation-based neoantigen approaches. Second, previous studies have revealed extensive de novo chromatin opening events in tumors, with these newly accessible regions often enriched for transcription factor binding sites.^55–57^ Our finding further demonstrates that HCC exhibits widespread specific open chromatin regions that are not only enriched with binding sites for transcription factors such as HNF4A-which shows a significant positive correlation with neoTST burden-but also activate cryptic regulatory elements (eg, TEs), leading to the production of numerous F-neoTSTs using novel TSSs. From a structural and functional perspective, neoTSTs exhibit multiple therapeutic implications: (1) membrane localization: Approximately 15% of neoTSTs contain conserved or de novo TMDs, facilitating efficient presentation via MHC class I/II molecules and enhancing T-cell recognition. This surface-exposed feature also makes them ideal targets for antibody-based therapies, such as bispecific antibodies or CAR-T. (2) Dual mechanisms of action: neoTSTs may directly modulate tumor biology through retained functional domains (eg, kinase domains), as illustrated in figure 4. These mechanisms include sustained activation of pro-proliferative signaling pathways (when kinase domains are preserved) or loss of tumor-suppressive functions (due to altered subcellular localization).

It is noteworthy that while current neoantigen research primarily focuses on antigens presented by HLA class I molecules and their role in activating CD8^+^ T cells, accumulating evidence highlights the critical function of HLA class II molecules and the CD4^+^ T cell responses they mediate in enhancing antitumor immunity.^58^ Through in-depth analysis of single-cell sequencing and RNA-seq data from immunotherapy patients, we observed a significant correlation between the activation status of CD4^+^ T cells and therapeutic efficacy, a process potentially under fine-tuned regulation by apCAFs. Animal studies further confirmed that neoTSTs not only effectively activate CD8^+^ T cells but also concurrently stimulate CD4^+^ T cells via the HLA class II pathway, thereby exerting synergistic antitumor effects. Although the accuracy of current HLA class II antigen prediction tools remains to be improved, our study employed a non-restrictive HLA-I screening strategy, which retains a broader spectrum of potential epitopes and effectively encompasses candidate targets likely presented through the HLA-II pathway, thus providing methodological support for comprehensively exploring the immunotherapeutic potential of neoTSTs. Animal experiments revealed that 60% of neoTSTs could simultaneously stimulate both CD4^+^ and CD8^+^ T cell responses. However, NetMHCpan predictions indicated that some of these neoTSTs lack predicted HLA-II binding motifs, further underscoring the necessity of a non-restrictive HLA screening approach. These findings not only expand our understanding of tumor immune response mechanisms but also provide a critical theoretical foundation for developing novel combination immunotherapy strategies that concurrently target both CD8^+^ and CD4^+^ T cells.

It is noteworthy that during the definition of neoTSTs, we categorized them into absolute and non-absolute subtypes, with the latter characterized by minimal expression in normal samples. This classification was intentionally designed to preserve potentially valuable targets that might otherwise be excluded. Sole reliance on the criterion of “complete absence in all normal samples” risks discarding tumor-enriched transcripts that exhibit trace-level detection in a minority of normal tissues due to technical sensitivity limits or biological heterogeneity. Moreover, transcripts that are highly expressed in tumors yet entirely undetectable in normal tissues also warrant careful evaluation as reliable biomarkers. Thus, our approach aims to balance specificity with the capacity for novel discovery.

While this study establishes a comprehensive HCC neoantigen repository and validates selected neoTSTs in preclinical models, several limitations should be acknowledged. First, although we confirmed the immunogenicity and antitumor activity of representative neoTSTs, the majority of candidates remain experimentally unverified, necessitating systematic functional characterization to assess their potential as shared therapeutic targets. Second, our immunopeptidomic validation, while informative, was constrained by the limited sample size and lack of paired tumor-normal data from existing datasets, highlighting the need for larger-scale, matched analyses. Although some neoTSTs contain TMDs, suggesting their membrane localization and high immunogenicity potential. However, this does not necessarily mean they will be eliminated in the immune editing process. Their persistent presence may be due to the tumor immune-suppressive microenvironment. The stable expression of these antigens makes them potential therapeutic targets to overcome immune evasion. In the future, their immunogenicity needs to be directly verified by detecting the T-cell and antibody responses of patients. Most importantly, current immunogenicity prediction tools exhibit marked bias toward MHC-I epitopes due to historical data limitations, while our findings demonstrate neoTSTs’ pronounced MHC-II immunogenicity-a critical but understudied aspect of antitumor immunity. These results underscore the urgent need for: (1) MHC-II-specific experimental validation platforms, and (2) development of balanced prediction algorithms incorporating both MHC-I and MHC-II immunogenicity data to fully exploit neoTSTs’ therapeutic potential.

## Conclusion

Our study established a comprehensive repository of potential neoTST candidates in liver cancer and elucidated their distinct splicing features and underlying drivers. These findings expand the understanding of neoantigen sources in liver cancer, providing a robust pool of high-potential targets that may enable shared therapeutic strategies. This resource offers critical support for enhancing liver cancer immunotherapy efficacy. Experimental validation-both in vitro and in vivo—confirmed the substantial immunotherapeutic potential of selected neoTSTs and their advantages in improving therapeutic outcomes. Collectively, this work holds considerable promise for broadening the clinical applicability of neoantigen-based immunotherapies.

## Supplementary material

10.1136/jitc-2026-015428 online supplemental file 1

## Data Availability

Data are available in a public, open access repository.
