# Supplementary material for: Decoding neoantigen-encoding tumor-specific transcripts unveils a shared target reservoir for immunotherapy in hepatocellular carcinoma
Source: J Immunother Cancer. 2026 Jul 21;14(7):e015428. doi: 10.1136/jitc-2026-015428 (PMC13405042; doi:10.1136/jitc-2026-015428)
Supplement: online supplemental file 1 [file jitc-14-7-s001.docx]

**Supplemental Materials for**

**Decoding Neoantigen-encoding Tumor-Specific Transcripts Unveils a Shared Target Reservoir for Immunotherapy in Hepatocellular Carcinoma**

Peng Lin, Yifan Wen, Jingjing Zhao, Feifei Zhang, Yaoming Su, Huiyi He, Hongwu Yu, Qiaojuan Li, Chengye Liu, Zhixiang Hu, Yan Li, Zhuting Fang, Linhui Liang, Shenglin Huang

Correspondence: Shenglin Huang (slhuang@fudan.edu.cn); Linhui Liang (lianglinhui@fudan.edu.cn); Zhuting Fang (ztfang@fjzlhospital.com)

This PDF file includes:

Materials and Methods

Supplemental Figure S1 to S7

Supplemental Tables S1 to S7

**Materials and Methods**

**Integration of multi-center liver cancer RNA-seq data**

To comprehensively characterize the transcriptomic landscape of liver cancer, we integrated RNA-seq data from 1,013 liver tissue samples, including hepatocellular carcinoma (HCC, n = 824), hepatoblastoma (HB, n = 124), and intrahepatic cholangiocarcinoma (ICC, n = 65). The data were aggregated from multiple public repositories and in-house sequencing efforts to ensure broad representation of molecular subtypes and clinical contexts. For HCC, 373 samples were obtained as BAM files from The Cancer Genome Atlas (TCGA-LIHC), 105 samples were sequenced internally (raw FASTQ files), and the remaining samples were compiled from 9 independent Gene Expression Omnibus (GEO) datasets (accession numbers: GSE112221, GSE144269, GSE94660, GSE114564, GSE124535, GSE140462, GSE148355, GSE77314, GSE77509). All 124 HB samples were curated from 7 GEO datasets (GSE104766, GSE133039, GSE151347, GSE81928, GSE89775), while ICC samples included 65 samples from 4 GEO datasets (GSE107943, GSE119336, GSE162396, GSE63420).

For non-TCGA samples, raw FASTQ files were processed using STAR (v2.5.3a) (1) for alignment to the GRCh38/hg38 p12 reference genome in two-pass mode with chimeric junction detection enabled, followed by duplicate marking with Picard Tools (version 2.23.3, https://broadinstitute.github.io/picard/). TCGA BAM files were converted to FASTQ using samtools bam2fq and reprocessed identically to ensure uniformity. Transcript abundance was quantified via StringTie (v2.2.1) (2) with GENCODE v29 annotations. This standardized pipeline enabled robust cross-cohort comparisons of liver cancer subtypes.

**Reference Transcriptome Dataset Construction**

To establish a comprehensive reference transcriptome dataset for comparative analysis, we integrated RNA sequencing (RNA-seq) data from over 20,000 samples, encompassing normal tissues, adjacent non-tumor tissues, and disease-associated liver tissues across 30 distinct tissue types. The dataset included 1,000 liver tissue samples, 600 adjacent non-tumor samples (of which 50 were paired with tumor samples from our internal cohort), and 20,000 samples from other normal tissues, primarily sourced from the Genotype-Tissue Expression (GTEx) project, The Cancer Genome Atlas (TCGA), and Gene Expression Omnibus (GEO) databases. To account for potential confounding effects of non-neoplastic liver diseases, we supplemented the reference with 779 RNA-seq profiles from non-cancer disease tissues (e.g., cirrhosis, hepatitis B/C), curated from 11 independent GEO datasets (accession numbers: GSE126848, GSE130970, GSE135251, GSE162694, GSE167523, GSE142530, GSE143318, GSE155907, GSE84346, GSE114564, GSE148355), 1 Array Express dataset: E-MTAB-6863 and 1 in-house data. This diverse dataset ensures robust background signals for tumor-specific analyses while controlling for inflammation- and fibrosis-related transcriptional changes. All data were uniformly processed using the pipeline described in the Integration of Multi-Center Liver Cancer RNA-seq Data section.

All tissue RNA-seq data were uniformly processed using StringTie (v2.2.1) for transcript assembly and quantification. Alternative splicing events were identified with ASJA (3), and expression levels were quantified as coverage per ten million reads (CPT). Subsequently, we constructed a comprehensive reference tissue database by generating 33 independent reference cohorts for each exon junction and single-exon transcript, derived from 30 normal tissue types, adjacent non-tumor tissues, and three liver disease sources. For each single-exon transcript, unique ID was confirmed based on their open reading frame (ORF). For each cohort, three key parameters were calculated: (a) median expression (CPT/TPM), (b) detection frequency, and (c) maximum expression value. This multi-dimensional reference framework enables robust normalization and context-specific analysis of splicing-derived neoantigens. TPM: Transcripts Per Million.

**Multi-exon tumor-specific transcripts detection**

Multi-exon tumor-specific transcripts were detected by identifying tumor-specific splicing. All of the transcript assemblies for each sample were obtained through StringTie. Junctions were identified and quantified using the ASJA tool, based on transcript assemblies. Junction expression was measured as coverage per ten million reads (CPT). When a junction was present in multiple transcripts, the transcript with the highest CPAT score and the highest transcript per million (TPM) value was selected as the representative isoform.

Considering that some tumor-enriched transcripts that can only be detected at very low levels in a few normal tissues might have high immunogenicity due to technical sensitivity limitations or biological heterogeneity, we have retained some transcripts with low background expression, considering them to be also tumor-specific transcripts. A splicing junction was classified as tumor-specific if it met at least one of the following criteria relative to the reference pool:

Absolute TST: Absolute tumor-specific: completely absent from all reference cohorts;

Non-Absolute TST: Low background prevalence: (1) the transcript must be detected in <5% of samples within any independent reference cohort; (2) it must show ≥4-fold higher expression in tumors compared to the mean expression level in the reference cohort; and (3) the tumor expression level must exceed twice the maximum value observed in the reference cohort.

The corresponding transcripts were selected through specific splicing. Transcripts with an expression value greater than 5 TPM are considered as tumor-specific transcripts, while the others are excluded.

**Single-exon tumor-specific transcripts detection**

The transcripts with expression values greater than 5 TPM were analyzed to identify single-exon tumor-specific transcripts. For each single-exon transcript, unique ID was confirmed based on their open reading frame (ORF) to enable appropriate comparison with reference cohort. ORF of each transcript was predicted using the CPAT (4) software. For each transcript, all possible ORFs were predicted, and the optimal ORF was selected as the ORF for that transcript. Transcripts with probability <= 0.7 or 5' UTR length > 200 were excluded. Single-exon tumor-specific transcripts were identified using the same criteria applied to the tumor-specific splicing.

**TST-derived neoantigens prediction module**

To predict TST-derived neoantigens, single-exon and multi-exon TSTs were first merged and filtered based on coding potential, as determined by CPAT (v0.1). Only those transcripts classified as coding by CPAT and containing a complete open reading frame (ORF) were retained as coding TSTs. To minimize false positives, only ORFs of coding TSTs directly resulting from tumor-specific transcriptional events (tumor-specific splicing) and single-exon TSTs were translated into protein sequences (in silico translation) by Biopython (v1.81) (5).

20,420 protein sequences were download from UnipProt (www.uniprot.org) as reference protein library. By comparing with the reference protein, the newly identified peptide segments encoded by each transcript were confirmed. For each new peptide segment, 11 amino acids were added at both its beginning and end, resulting in the candidate peptide segment used for predicting potential antigenic epitopes. If the new peptide is at the N-terminus or C-terminus of the protein, then only extend one side of it. HLA class I genotyping was performed using arcasHLA (v3.9) (6) for samples from GEO and in-house data. HLA typing results for TCGA-LIHC samples were obtained from the GDC (see Prediction HLA Typing section). Only HLA alleles supported by NetMHCpan were retained. In total, high-confidence HLA genotypes were determined for 1,048 samples.

NetMHCpan (v4.1) (7) was used to predict 8-12 amino acid peptides binding to HLA, using the parameters ‘-f -inptype 0 -BA -xls -a’. Peptides with predicted binding affinity scores (IC50) <500 nM were classified as either strong binders (SB) or weak binders (WB) and considered candidate neoantigens. Final TST-derived neoantigens were defined as peptides absent in reference protein library.

**ESTIMATE Immune Score**

**We calculate the ESTIMATE immune score by using ESTIMATE v1.0.13.** The ESTIMATE (Estimation of Stromal and Immune cells in Malignant Tumor tissues using Expression data) algorithm infers the immune score and stromal score from te overall gene expression data of tumor tissues by leveraging the characteristic expression profiles of specific gene sets that are highly correlated with the abundance of immune cells and stromal cells. These scores are continuous numerical values, with higher values indicating a higher estimated abundance of the corresponding component (immune or stromal cells) in the tumor microenvironment.

**Proteomic analysis and Immunopeptidomes data analysis**

All the Neoantigen-encoding TSTs (neoTSTs) of each tumor sample were translated into protein sequences using Biopython (v1.81) to generate sample-specific protein databases. Protein sequences were merged and deduplicated and then create custom protein and decoy databases with the philosopher database --custom command.

HCC mass spectrometry data PDC000198 (n = 171) were download from Proteomic Data Commons (PDC). The spectrum files were analyzed using matched custom protein and decoy databases. Raw mass spectrometry data were converted to mzML format using ThermoRawFileParser (v1.4.2). Searches were performed using MSFragger (v3.8) (8) and the Philosopher (v5.0) workflow with the following parameters: precursor mass tolerance of ±10, fragment mass tolerance of ±20 ppm, and enzyme cleavage settings defined in the closed_fragger configuration file provided by MSFragger. Methionine oxidation (+15.994915) and serine TMT labeling (+229.162932) were specified as variable modifications, while cysteine carbamidomethylation (+57.021464) and lysine TMT labeling (+229.162932) were set as fixed modifications. Proteins were filtered with Philosopher using an FDR threshold of <0.01.

Immunopeptidome PXD023143 of HCC were download from Proteome Xchange (proteomecentral.proteomexchange.org), including 8 samples. Raw data were converted to mzML format and analyzed using Comet software within the MSFragger and Philosopher. Precursor tolerance was set to ±10 ppm, and fragment ion m/z tolerance was set to ±10 ppm. Peptides ranging from 8 to 11 amino acids were searched using "unspecific" digestion parameters. Peptides and protein were filtered with Philosopher using an FDR threshold of <0.01.

**Splicing junction classification**

Splicing junctions were categorized using a two-step hierarchical approach with bedtools intersect (chain-specific -s parameter). First, each junction was annotated by comparing with GENCODE features to generate composite labels (e.g., Exonic (E), Intronic (In), Intergenic (Ig), or Known junction (J)), where concatenated labels like InJ indicated intronic donor with known acceptor junction. Known junctions (both donor/acceptor = "J") were subclassified into Annotated Junctions (AJ) (existing in GENCODE) or Novel Junctions (NeoJ) (de novo identified). Based on transcript structural impacts, junctions were further grouped into five categories: exon skipping (internal exonic segments skipped, *NeoJ*), exon truncation (partial exon shortening, *E, InE, IgE, EJ*), intron retention (partial intronic sequence retention, *In, InE, InIg, InJ*), intergenic retention (partial intergenic region incorporation, *Ig, IgE, InIg, IgJ*), and Annotated Junctions (AJ).

**Transcript encoding potential and protein sequence prediction**

Transcripts coding potential was assessed using CPAT (v3.0.5; human model, cutoff > 0.7). Transcripts were classified as protein-coding only when both tools yielded concordant results and the sequences contained canonical start (ATG) and stop codons (TAA, TAG, or TGA). The translate package of Python was used to convert the mRNA sequence into the protein sequence.

**Annotation of neoPep position**

The pickle package in Python was used to convert the 20,420 reviewed protein sequence files in UniProt into hash files with an amino acid length of 8. Compare the protein sequence translated from neoTST with the hash file. If there is a peptide segment of 8-mers in neoTST that is not present in the hash file, then keep it as neoPep. Finally, for each neoPep in the neoTST, annotations are made based on its position relative to the protein sequence. If the positions are adjacent, they are concatenated.

**NeoTST classification**

Chimeric-neoTS: To identify chimeric transcripts, we performed exon-level comparisons between neoTSTs and known exons using bedtools intersect -s -wo. A neoTST was classified as chimeric if it contained at least one exon that was 100% identical to any annotated exon in the reference genome, suggesting potential exon recombination events.

F-neoTST: For N-terminal neoantigen identification, we defined F-neoTSTs as transcripts where the starting position of the predicted neoepitope aligned with the first amino acid of the protein sequence. In cases where multiple neoepitopes were predicted, the earliest starting position was used as the primary determinant for classification, ensuring conservative annotation of N-terminal neoantigens.

TE-neoTST: Transposable element-associated neoTSTs were identified by intersecting the first exons of neoTSTs with annotated TE regions from the UCSC RepeatMasker database (hg38 assembly). Using bedtools intersect -s -wo, we classified any neoTST as TE-associated (TE-neoTST) if its first exon showed genomic overlap with any of the >5 million annotated TE positions, indicating potential TE-mediated transcriptional initiation.

**Regulatory analysis of F-neoTSTs**

Homer was used to predict the transcription factors that are enriched in the TSS regions (±1000 bp) of the F-neoTST. The ATAC-seq data are derived from internal sources and are used to analyze the chromatin openness of the regions where HNF4A binds.

**Identification of sc-neoTST in scRNA-seq**

Cellular cluster-level neoTST quantification: Single-cell clustering was performed using CellRanger (v6.0.0) and Seurat (v4.0.5) to generate annotated cell clusters (see Methods: Single-cell RNA Sequencing Analysis). For each cluster, we aggregated single-cell BAM files after removing cell barcodes to create pseudo-bulk samples. Following bulk RNA-seq analytical pipelines, we performed transcriptome assembly using StringTie and quantified splice junctions with ASJA. Cluster-specific neoTSTs were identified by comparing tumor clusters against both normal single-cell data and previously annotated neoTSTs from bulk analyses.

Single-cell resolution neoTST detection: The SCASL pipeline was employed for single-cell splice junction analysis, focusing on cells expressing >2,000 genes to ensure data quality. For tumor samples, we extracted individual cell BAM files and identified splice junctions using SCASL's extract.py function (adjacent tissues were processed as whole samples). neoTST candidates were stringently filtered by: (1) removing junctions detected in matched adjacent tissues, (2) excluding junctions present in our reference database, (3) eliminating junctions found only in non-tumor cells from the same sample, and (4) retaining only junctions overlapping with our bulk-derived neoTST catalog. This multi-step verification ensured high-confidence single-cell neoTST identification. For a neoTST transcript to be considered “detected” in a particular single cell, we require that it has at least 3 reads that support the connection of the characteristic splicing site of the neoTST. For neoTSTs defined by multiple splicing junctions, as long as any one of the characteristic junctions is detected in the cell, this neoTST will be included in the count of the cell. This enhances the sensitivity for detecting at low abundances while ensuring that the transcribed molecules counted are truly related to the neoTST.

**Prediction HLA Typing**

For TCGA-LIHC samples, we utilized HLA typing results predicted by OptiType (9), which were downloaded from the GDC. Additionally, HLA class I alleles were inferred using arcasHLA (v3.9) for other samples. RNA-seq BAM files were processed through the sequential extract, genotype, partial, and merge modules of arcasHLA to generate final MHC class I haplotypes. To ensure compatibility with downstream neoantigen binding predictions using NetMHCpan, only HLA alleles supported by NetMHCpan were retained. Following this comprehensive analysis, HLA types were successfully determined for a total of 1048 samples.

**Identification of neoMut**

For TCGA-LIHC samples, we obtained pre-curated SNV-derived neoantigens from the Tumor-Specific Neoantigen Database (TSNAdb) (10). To ensure data reliability, we performed cross-validation by retrieving raw somatic mutation profiles and HLA typing data from the GDC portal and comparing them against TSNAdb's neoantigen predictions. This two-source verification approach enhanced the accuracy of our mutational neoantigen dataset.

For samples with whole-exome sequencing (WES) data, we selected mismatch mutations meeting stringent quality thresholds (sequencing depth >5, TPM >10) for neoantigen prediction. In WES-unavailable cases, we identified somatic mutations from RNA-seq data using GATK, followed by comparison with normal samples to exclude germline variants. SNV effect analysis was performed using snpEff to derive mutated protein sequences, from which we extracted 23-mer peptides (mutated residue ±11 flanking amino acids) to ensure proper MHC binding context (reference: human-GRCh38, mouse-GRCm38 from https://useast.ensembl.org/). All candidates were predicted using NetMHCpan (v4.1) with consistent parameters (binding affinity cutoff, peptide length) as applied to TST-derived neoantigens. Finally, we filtered out any peptides matching the human reference proteome to retain strictly tumor-specific neoantigens.

**Survival analysis and differential gene expression**

Kaplan-Meier analysis was performed to assess the relationship between neoTST burden (number) and neoMut burden (number) with overall survival (OS). The load levels are classified based on the average value of the loads. Those above the average are defined as the high-load group. The survfit and Surv functions from the survival package were used, and survival curves were visualized using the ggsurvplot function. The surv_cutpoint function was applied to determine the optimal cutoff for survival grouping. Similarly, the expression data (TPM) of HNF4A was collected. Again, based on the average values, the patients were divided into the high HNF4A group and the low HNF4A group according to the levels of HNF4A expression.

**Representative neoTSTs of HCC selection and experimental validation**

Twenty neoTSTs resulting from retention events in non-coding regions have been proven to have a wide distribution. Based on HLA subtypes prediction, the neoTST containing HLA-A:11*01 or HLA-A:02*01 was retained for experimental verification. In vivo immunogenicity testing was performed in B6-hHLA-A11:01/hB2M (Strain No. T064359) and B6-hHLA-A02:01/hB2M (Strain No. T064344) transgenic mice (8-10 weeks old, GemPharmatech, China). Coding sequences of selected neoTSTs containing predicted neoantigens were synthesized, cloned into a mRNA expression vector, and polyadenylated to generate mRNA templates. These were used for in vitro transcription (IVT) with N¹-methylpseudouridine modification using the T7 High Yield RNA Synthesis Kit (Yeasen, 10633ES60). To formulate lipid nanoparticle (LNP)-encapsulated mRNA vaccines (mRNA-LNP), SM-102 ionizable lipid, cholesterol, DSPC, and DMG-PEG2000 were dissolved in ethanol and mixed with mRNA in 100 mM citrate buffer (pH 4.0) at a 3:1 volume ratio (ethanol:aqueous phases) using the INano™ L microfluidic mixer (Micro&Nano). After concentration and purification, the resulting mRNA-LNP formulations were obtained. Fourteen HCC neoTSTs were randomly divided into 3 pools (5 µg mRNA each) and formulated into two mRNA-LNP vaccines. Humanized HLA-A:02*01/HLA-A:02*01 mice received intramuscular (IM) injections at two separate anatomical sites during the first and second weeks, respectively. In the third week, mice were euthanized, and splenocytes were isolated for immunological assessment by IFN-γ ELISpot and flow cytometry (see Flow cytometry analysis and IFN-γ ELISpot assay section).

**Confocal Microscopy for Subcellular Localization of TMD-neoTSTs**

In order to identify the subcellular localization of TMD-neoTSTs, the EGFP-tagged neoTST was made by cloning synthesized sequences into pCDH-CMV-MCS-EF1-Puro vector using EcoRI and BamHI. The EGFP was served as the negative control. Subsequently, the lentiviral particles were generated by HEK293T cells, which were transfected with the above lentiviral vectors (2 μg), packaging plasmid (psPAX2, 1.8μg), and VSV-G envelope plasmid (pMD2.G, 0.8 μg) DNAs. 8 hrs later, the medium was exchanged. The virus particle-containing medium were harvested after 2 days and filtered and stored in -80 °C. The stable expression of TMD-neoTSTs cell line constructed in HEK293T cells, which were transduced by combining 1 ml of viral particle-containing medium with 8 mg/ml polybrene and selected by using 2 mg/mL puromycin after 48 hrs transduction. For cell imaging, cells were seeding on coverslips for about 24 h and fixed in 4% PFA for 10 min at room temperature. After washing twice in PBS, cell membrane was marked using a red fluorescent probe DiD Perchlorate (DiIC_18_(5), Yeasen), wherein DiD was added to serum-free medium at a final concentration of 5 μM for 15 min at 37 °C. At the end of the incubation period, the cells were rinsed three times in PBS and then stained the nuclei with DAPI (Beyotime, China) for 20 min at room temperature. Images were captured using a Olympus FV3000 confocal microscope (Olympus, Japan).

**Hep53.4 neoTST identification**

We collected 265 RNA-seq datasets from mouse liver and other normal tissues as controls, organizing them into two reference cohorts following the same methodology used for human samples. The neoTST identification process for Hep53.4 cells mirrored our human analytical pipeline, while Hep53.4-derived TSTs were consolidated and their protein-coding sequences extracted for subsequent neoantigen prediction. Coding potential was assessed using CPAT with mouse-specific genome annotations (probability threshold >0.44), maintaining all other parameters consistent with human TST analysis. For neoantigen prediction, we employed NetMHCpan (v4.1) to screen 8-11-mer peptides binding to murine MHC class I (H-2-Db and H-2-Kb), applying identical selection criteria as in human studies. To ensure tumor specificity, we excluded all peptides matching our comprehensive reference database, retaining only truly novel TST-derived neoantigens.

**Hep53.4 neoMut identification**

Three complementary variant callers were employed for comprehensive mutation detection from RNA-seq data: GATK HaplotypeCaller (v4.2.2.0), Strelka2 (v2.9.2), and Samtools (v1.6). STAR-aligned BAM files underwent rigorous preprocessing using Picard (v2.23.3) and GATK tools, sequentially executing: CleanSam, AddOrReplaceReadGroups, MarkDuplicates, SplitNCigarReads, BaseRecalibrator, and ApplyBQSR. Variant calling with HaplotypeCaller was followed by quality filtering (FS >30.0, QD <2.0, DP <10, AD <5), while Strelka2 outputs were filtered for 'PASS' variants. Samtools analysis incorporated flagstat assessment before mpileup variant calling with bcftools, all using default parameters.

We required consensus detection by ≥2 callers to designate NeoMut candidates, then applied additional filters: sequencing depth >20, mutant allele depth >5, and gene expression >10 TPM. Germline variant exclusion was achieved by constructing a normal tissue mutation profile from 8 control samples (SRR20283751.), retaining only Hep53.4-specific mutations. This multi-layered approach ensured high-confidence somatic mutation identification.

Non-synonymous mutations (ANNOVAR-annotated) were processed through: (1) snpEff-derived protein sequence alteration; (2) MHC binding prediction via NetMHCpan (v4.1; 8-11 mers for H-2-Db/Kb) and NetMHCIIpan (v4.1; 14-18mers for H-2-IAb), using extended flanking sequences (11 aa for class I, 25 aa for class II). Peptides were classified by binding affinity (SB: < 500nM, WB: ≥ 500nM) and filtered against the mouse proteome to ensure tumor specificity. This comprehensive workflow maximized neoantigen prediction reliability while minimizing false positives.

**Cell Cultures**

The murine hepatocellular carcinoma cell line Hep-53.4 (BioVector NTCC Plasmid Vector Microbial Culture Collection), derived from a C57BL/6J mouse model of liver cancer, was cultured in Dulbecco's Modified Eagle Medium (DMEM; Basalmedia, L110KJ) supplemented with 10% fetal bovine serum (FBS; BDBIO, F814-500) and 1% penicillin-streptomycin (P/S; Basalmedia, S110JV). Splenocytes were maintained in RPMI 1640 medium (Basalmedia, L210KJ) containing 4 ng/ml GM-CSF (PeproTech, 315-03-20UG), 10% FBS, and 1% P/S. All cells were incubated at 37°C in a 10% CO₂ atmosphere and routinely tested negative for mycoplasma contamination prior to experimentation.

**Animal model and experimental design**

Six- to eight-week-old male C57BL/6 mice (Shanghai Jihui Laboratory Animal Care Co., Ltd.) and genetically modified strains (B6-hHLAA11.1/hB2M and B6-hHLAA2.1/hB2M; GemPharmatech) were used in this study. To establish Hep-53.4 subcutaneous tumors, 2.5×10⁵ cells suspended in 200 μL serum-free DMEM were injected into the right dorsal flank. On day 3 post-inoculation, mice were randomly allocated to three treatment groups: (1) Control: 100 μL PBS administered intramuscularly (i.m.) every 7 days (2 doses total); (2) NeoTSTs: 5 μg neoTST mRNA-LNP (i.m., 100 μL) every 7 days (2 doses); (3) NeoMuts: 5 μg neoMut mRNA-LNP (i.m., 100 μL) every 7 days (2 doses). Tumor dimensions were measured every 2–3 days using digital calipers, with volumes calculated as V = (L × W²)/2. A strict endpoint criterion (tumor volume >1,500 mm³ or 20 mm in any dimension) was enforced. Mice were housed under specific pathogen-free conditions (12-h light/dark cycle, 20–22°C, 40–60% humidity). All procedures complied with Shanghai Laboratory Animal Care Association guidelines and were approved by the Fudan University Institutional Animal Care and Use Committee (IACUC protocol: 202510FD0002).

**In vitro Synthesis of Neoantigen mRNA**

The neoantigen peptide sequences were first converted to optimized nucleotide sequences using the NeoDesign (11) prediction algorithm. Full-length DNA templates were synthesized through a multi-round PCR amplification process. PCR products were purified using the FastPure Gel DNA Extraction Mini Kit (Vazyme, DC301-01) to obtain template DNA for in vitro transcription (IVT), with all templates validated by Sanger sequencing (GENEWIZ).

For mRNA production, IVT was performed using the T7 High Yield RNA Synthesis Kit (Yeasen, 10633ES60) at 37°C for 2 hours to generate N1-methylpseudouridine-modified mRNA. Template DNA was subsequently removed by DNase I treatment (Yeasen, 10325ES80, 37°C for 20 min), followed by column-based purification (GENSTONE BIOTECH, TR1017) to obtain high-quality mRNA products.

**Lipid Nanoparticle Synthesis and mRNA Encapsulation**

The lipid nanoparticles (LNPs) were formulated with SM-102 ionizable lipid, DSPC (structural lipid), cholesterol, and DMG-PEG2000 (PEGylated lipid) at a molar ratio of 50:10:38.5:1.5. The lipid mixture was first dissolved in ethanol, then combined with mRNA solution (in 100 mM citrate buffer, pH 4.0) at a 3:1 volumetric ratio (ethanol:aqueous phase) using an INano™L microfluidic mixer (Micro&Nano) for precise nanoprecipitation. Post-formulation, the crude LNP solution was concentrated and purified through 100 kDa molecular weight cutoff (MWCO) ultrafiltration (Millipore UFC510096). Encapsulation efficiency was quantified using the Equalbit RNA HS Assay Kit (Vazyme, EQ211-01) with or without 2% Triton X-100 (Sigma, T8787) treatment to differentiate encapsulated versus free mRNA (12).

**Flow cytometry analysis**

Splenocytes were isolated by homogenizing mouse spleens through a 70 µm cell strainer (Shanghai Biotend, BE4021), followed by red blood cell lysis using 1× RBC Lysis Buffer (BioGems, 64010-00) for 10 minutes. The isolated cells were seeded in U-bottom 96-well plates and transfected with 500 ng of neoantigen-encoding mRNA per well using CALNP™ mRNA transfection reagent (D-Nano, DN002-10) for 16 hours. Five hours prior to harvesting, Brefeldin A (Yeasen, 50504ES08) and Monensin (Yeasen, 50501ES03) were added to block cytokine secretion. Cells were then collected and stained with Fixable Viability Dye eFluor™ 450 (eBioscience, 65-0863-14) to distinguish live cells. Surface staining was performed using APC anti-mouse CD3 (Proteintech, APC-65077) and FITC anti-mouse CD8 (Proteintech, FITC-65069). Subsequently, cells were fixed and permeabilized using an intracellular staining buffer (eBioscience), followed by intracellular staining with PE/Cyanine7 anti-mouse CD4 (BioLegend, 100528) and PE anti-mouse IFN-γ (BioLegend, 505808) for 30 minutes at 4°C. Samples were acquired on a CytoFLEX flow cytometer (Beckman Coulter) for analysis.

**IFN-γ ELISpot Assay**

T cell activation was assessed using a Mouse IFN-γ Precoated ELISpot Kit (DAKEWE, 2210002). Splenocytes (5 × 10⁵ cells/well) were transfected with 100 ng of neoantigen-encoding mRNA per well using CALNP™ mRNA Transfection Reagent, while control groups received non-specific mRNA. After 24-hour incubation, plates were washed (per manufacturer’s protocol), followed by sequential addition of biotinylated detection antibody and streptavidin-HRP conjugate (1-hour incubation at 37°C). AEC substrate was then applied for spot development (15–30 min, RT), with reactions terminated by deionized water washing. Spot quantification was performed by Beijing Xingjianya Biotechnology Co., Ltd. using an automated ELISpot reader.

**Multiplex immunofluorescence (mIF) analysis**

Mouse tumor tissues were fixed, paraffin-embedded, and sectioned for further analysis.We performed mIF using antibodies against Col3a1 (Servicebio, GB111629-100), Cd74 (Servicebio, GB115175-100), and DAPI (Servicebio; G1012-10ML). Slides were scanned using Phenochart 2.0.1, and Formalin-Fixed Paraffin-Embedded (FFPE) slides were analyzed using Saiviewer 2.2.1.

**Statistical analyses**

Statistical analyses were performed using R (v4.0.2) and python (python 3.9.18). Comparisons between two groups were assessed by the Wilcoxon rank-sum test. Survival analyses utilized Kaplan-Meier estimators with log-rank tests to determine statistical significance. One-way ANOVA was employed for group comparisons in tumor size and flow cytometry data; results are presented as mean±SEM. Significance levels were defined as follows: *P < 0.05, **P < 0.01, ***P < 0.001.

**Supplemental Figure**


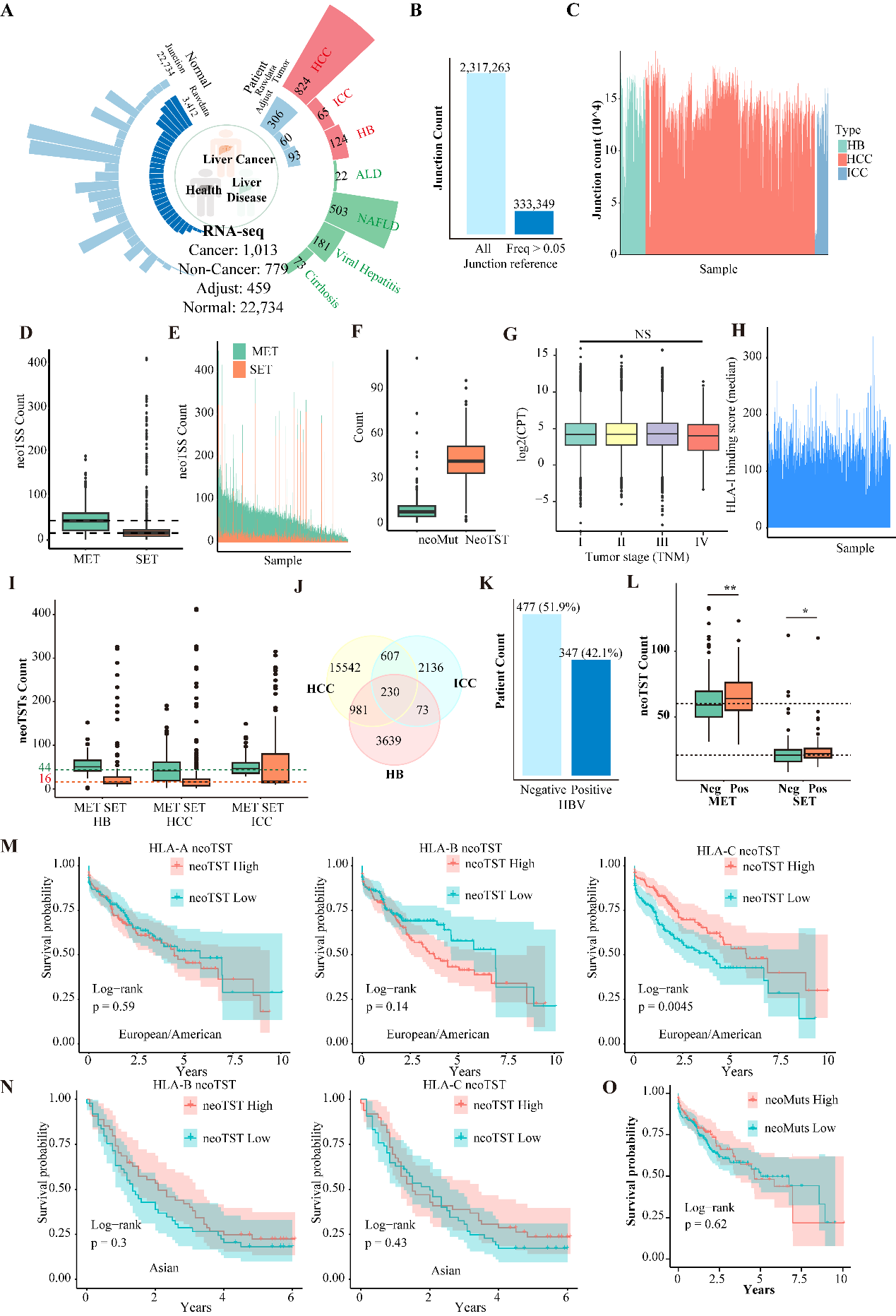


**Supplemental Figure S1. Landscape of Neoantigen-encoding tumor-specific transcripts (neoTSTs) in 1,013 Liver Cancer.** (A) Bar plot provides a comprehensive breakdown of the distribution of junctions numbers in the biological samples analyzed in the study. These samples were classified according to their tissue origin and disease status. (B) Quantifies splice junction of controls from raw BAM files (left, n > 3,000) and junction frequency threshold: ≥5% occurrence in any GTEX tissue (right, n > 20,000). (C) Total number of RNA splicing junctions detected per tumor sample, stratified by molecular subtypes of liver cancer. (D) Boxplot shows count of MET and SET. (E) Bar plot provides a comprehensive breakdown of the distribution of neoTSTs (MET and SET) numbers in the biological samples analyzed in the study. (F) Boxplot shows count of neoMuts and neoTSTs. (G) The box plot shows the expression of neoTSTs in different tumor stages. The x-axis represents different stages, and the y-axis represents log2(CPT), which is the standardized expression value of tumor-specific splicing sites. Statistical significance was determined by Wilcoxon rank-sum test (NS: p > 0.05). (H) Bar plot provides a comprehensive breakdown of the distribution of median HLA binding score in the biological samples analyzed in the study. (I) Prevalence of multi-exonic transcripts (METs) and single-exonic transcripts (SETs) count in different cancer types. X-axis: Cancer types (HB, HCC, ICC). Y-axis: neoTSTs count. Orange dashed: Mean SETs frequency (16 events/sample). Green dashed: Mean MET alteration frequency (44 events/sample). (J) Venn diagram quantifies neoTSTs identified in 3 major liver cancer types, revealing both shared and specific neoTSTs count. (K) Prevalence of hepatitis B virus (HBV) infection in the study's HCC samples. (L) Count of neoTSTs between HBV-positive and HBV-negative HCC patients. Statistical significance was determined by Wilcoxon rank-sum test (***p < 0.001). (M) Kaplan-Meier survival analysis stratified by neoTST burden (high vs. low) of different HLA subtype (HLA-A, HLA-B, HLA-C) in TCGA-LIHC cohort. Log-rank test was used. The average value of the neoTSTs burden was used as the cutoff point. (N) Kaplan-Meier survival analysis stratified by neoTST burden (high vs. low) of different HLA subtype (HLA-B, HLA-C) in in-house cohort. Log-rank test was used. The average value of the neoTSTs burden was used as the cutoff point. (O) Kaplan-Meier survival curves for the TCGA-LIHC cohort stratified by neoMuts burden. The mean neoMut burden was used for stratification.


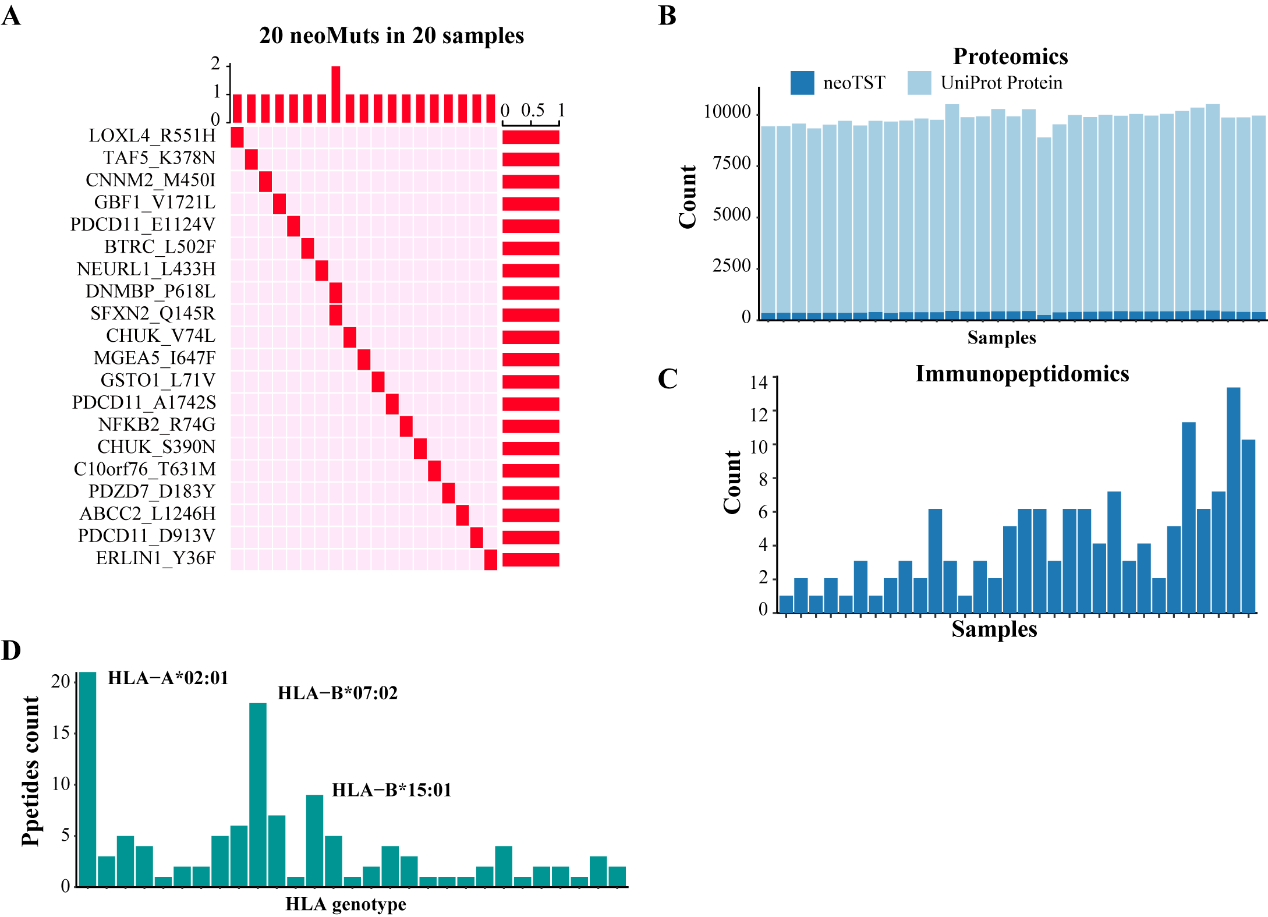


**Supplemental Figure S2. Mass Spectrometry and Immunopeptidomics-Based Identification and Immunogenicity Assessment of neoTSTs in Liver Cancer.**

(A) Distribution pattern of the 20 neoMuts across HCC samples from TCGA-LIHC. (B) Proteomic identification of neoTSTs versus canonical proteins in HCC. (C) Immunopeptidomic identification of neoTSTs in HCC. (D) The number of different HLA subtypes identified by the neoTSTs in the immunopeptide spectrum. The HLA subtypes with the highest frequencies are highlighted.


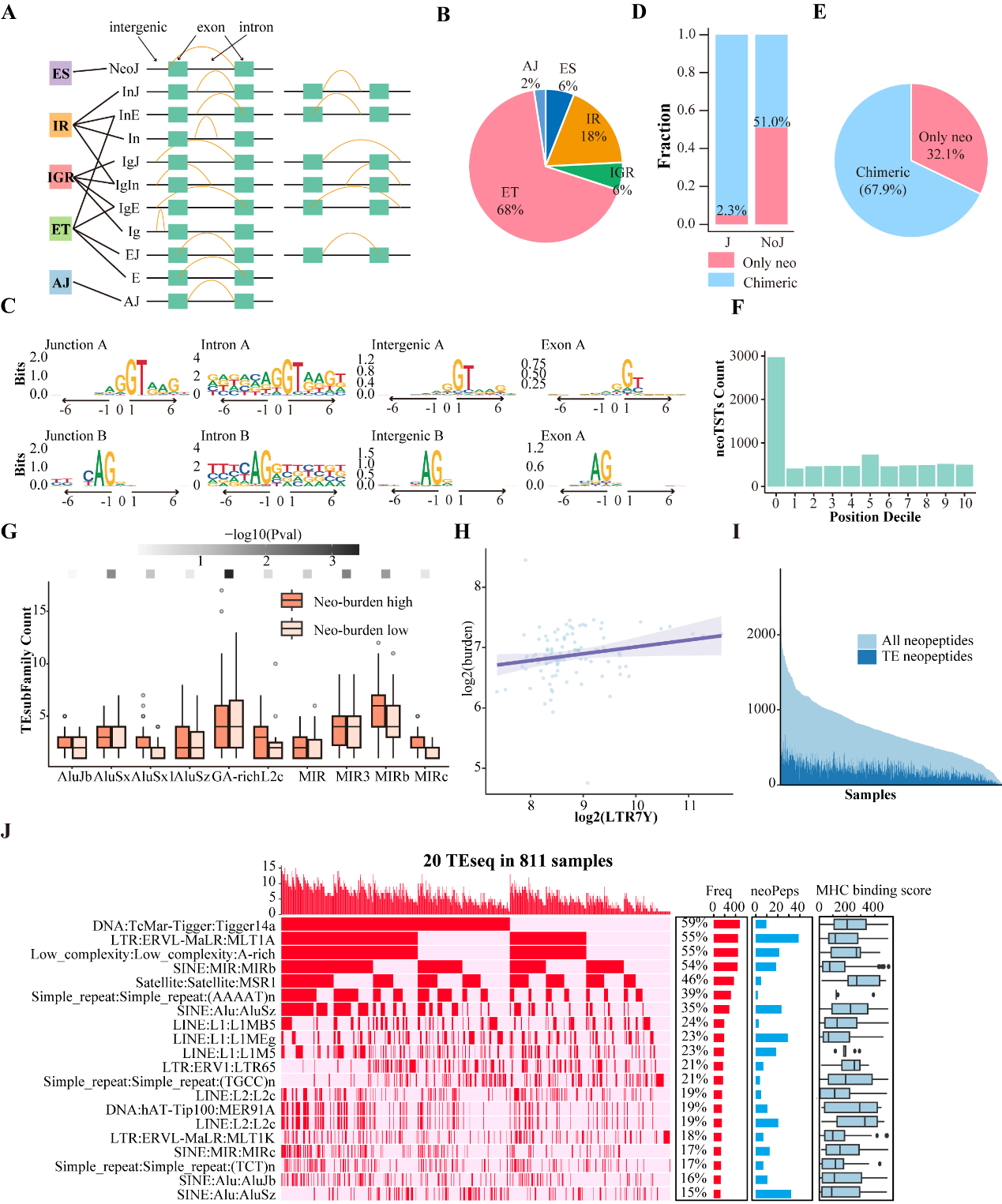


**Supplemental Figure S3. Retention of noncoding sequences and transposon-driven activation generate high-yield neoantigen expansion.** (A-B) Classification and proportions of splicing junction types. (C) Distribution of splicing signals from different sources. (D-E) Proportions of Chimeric-neoTSTs and nonchimeric-neoTSTs driven by J or neoJ. (F) Examines the spatial distribution of predicted neoantigen-peptides (neoPeps) across tumor-specific transcript isoforms (neoTSTs) based on their protein sequence positions. Here, 0 indicates that neoPep is located at the beginning of the neoTST protein. 1-10 indicate that neoPep is located at the n/10 region of the neoTST protein. (G) The boxplot compares the expression levels of individual TE subfamilies between high neoTSTs burden samples and low neoTSTs burden samples. Each box represents the distribution of subfamily expression within a TE type. Dots indicate outliers. (H) Scatter plot showing the positive correlation between log2(LTR7Y expression) (x-axis) and neoTST burden (y-axis, count per sample). A trendline (blue dashed line) indicates a significant linear relationship (r = 0.27, p < 0.001). (I) The quantity of neoPeps derived from TE. (J) The frequencies of the top 20 TE-neoTST distributions and the frequencies, quantities, and MHC affinity scores of the resulting neoPeps.


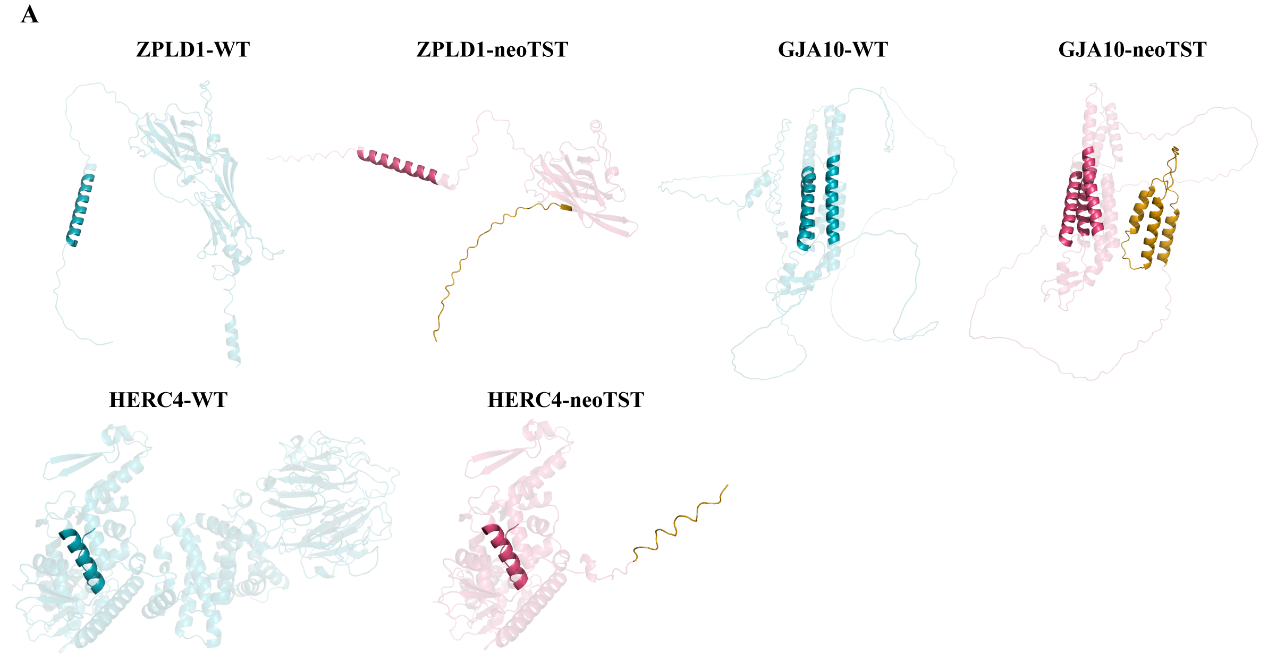


**Supplemental Figure S4. neoTSTs could generate de novo transmembrane domains (TMDs).** (A) The figure presents the 3D structural models of: Wild-type ZPLD1 (blue ribbon diagram) ZPLD1-neoTST (red ribbon diagram); Wild-type GJA10 (blue ribbon diagram) GJA10-neoTST (red ribbon diagram); Wild-type HERC4 (blue ribbon diagram) HERC4-neoTST (red ribbon diagram).


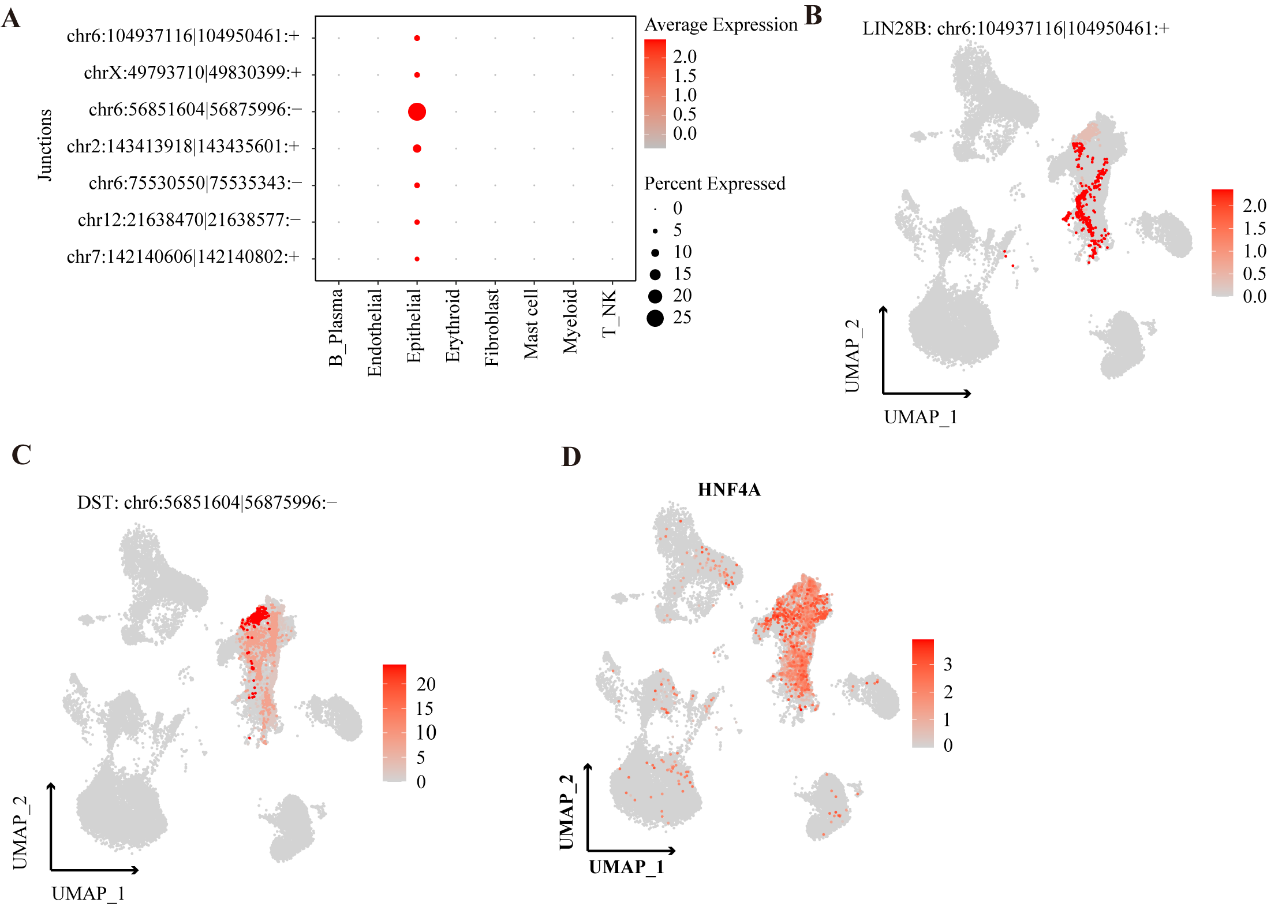


**Supplemental Figure S5. HNF4A binding activates neoTSTs transcription in HCC.** (A) Dot plot shows the expression of specific splicing sites in different cells. (B-C) UMAP plot shows the distribution of LIN28B-neoTST and DST-neoTST in different cells. (D) UMAP plot highlights HNF4A expression within the single-cell landscape of liver cancer.


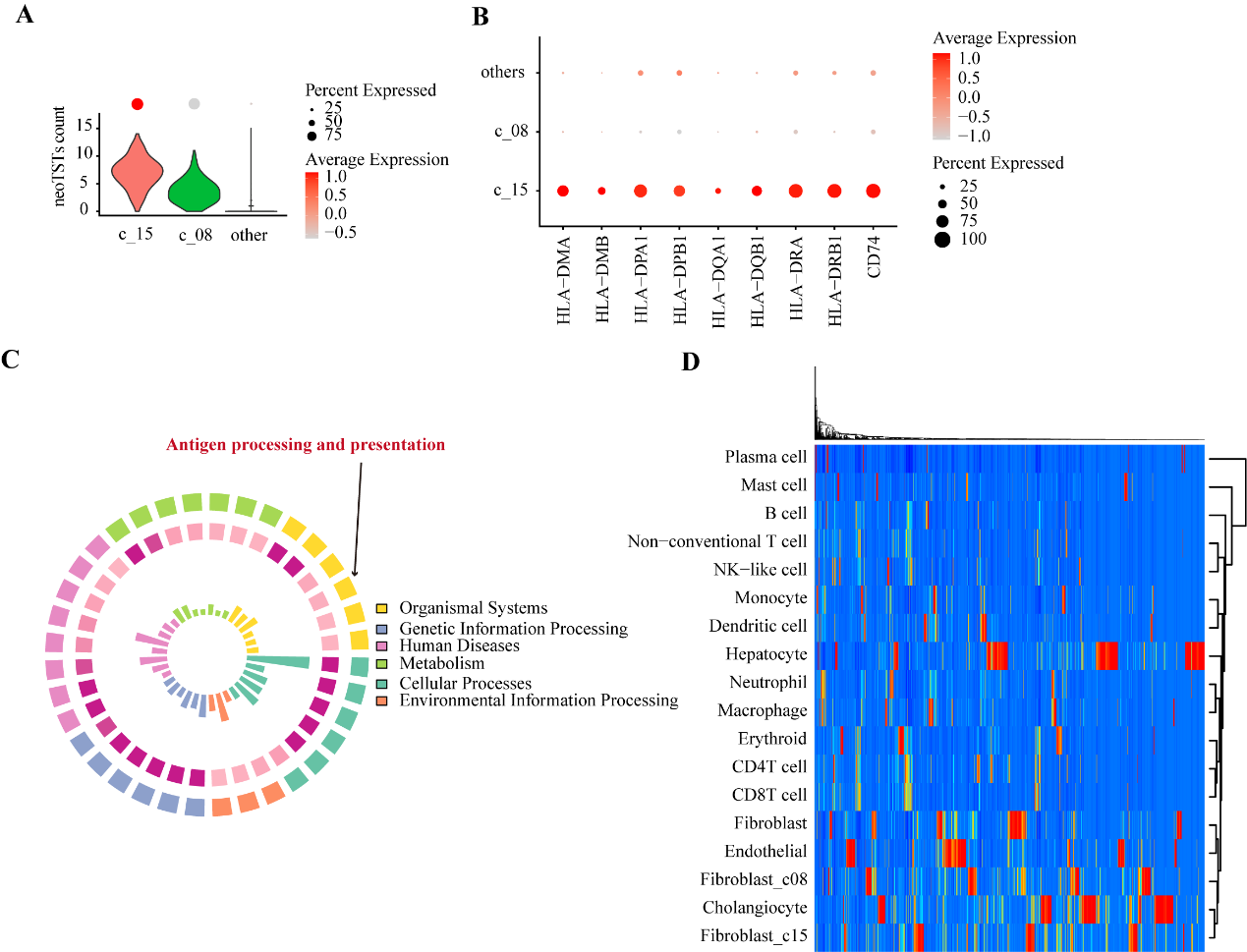


**Supplemental Figure S6. Heterogeneity of neoTSTs at single-cell resolution.** (A) Count of neoTST in C_15, C_08 and other cancer-associated fibroblasts (CAFs). (B) Signatures expression of antigen-presenting CAF (apCAF) in C_15, C_08 and other CAFs. (C) KEGG enrichment results of up regulated genes in C_15, only highlights and marks the antigen processing and presentation pathway. (D) Heatmap shows the expression profiles of canonical marker genes across distinct cell populations.


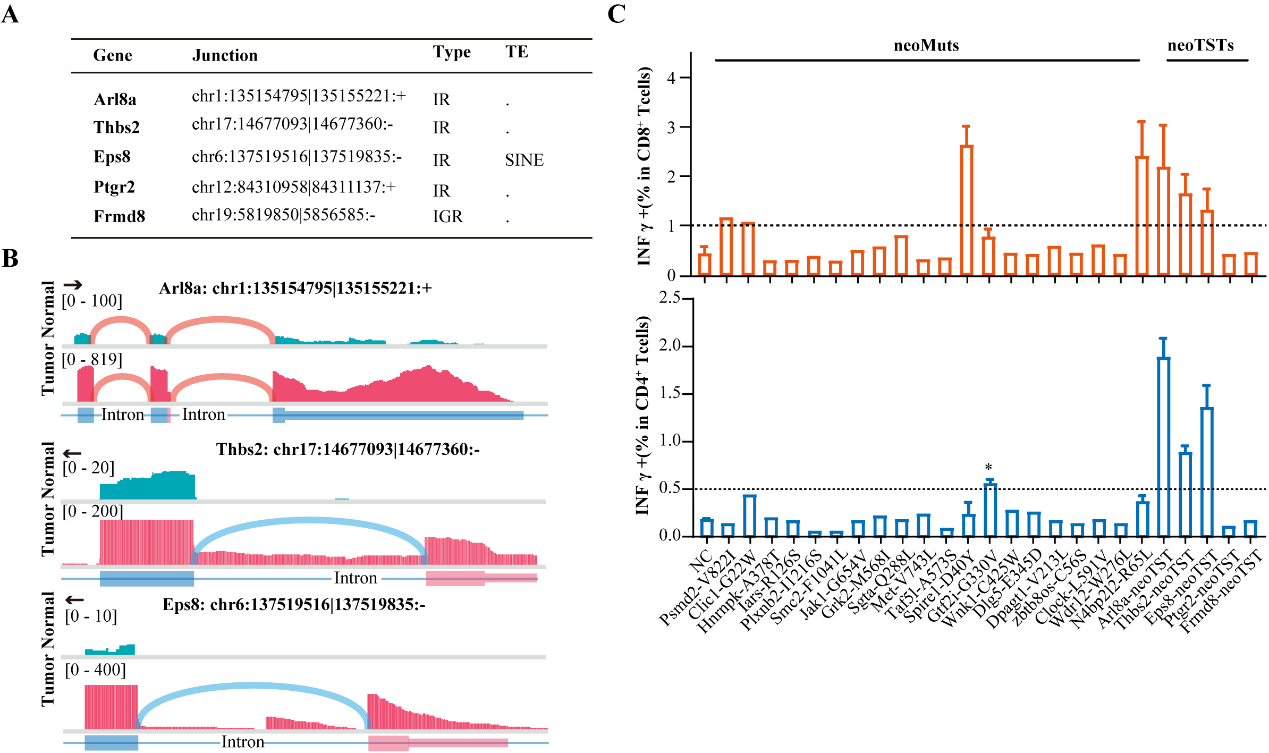


**Supplemental Figure S7. neoTST elicited antigen-specific CD8+ & CD4+ T cell responses and inhibited tumor growth in a syngeneic HCC model.** (A) Characterization of representative neoTSTs in Hep53.4. (B) Sashimi plot of 3 neoTSTs in Hep53.4: Arl8a-neoTST, Thbs2-neoTST and Eps8-neoTST. (C) Flow cytometry results of 20 neoMut and 5 neoTST vaccines.

**Supplemental Tables S1. 13 neoTSTs for experimental verification.**

| Junction | gene | type | position | HLA-A*02:01 | HLA-A*11:01 |
| --- | --- | --- | --- | --- | --- |
| chr6:104937116\|104950461:+ | LIN28B | IR | 1 | . | SSAPGGASK MSHRRQVLQK QVSSAPGGASK VSSAPGGASK |
| chr3:102457662\|102457781:+ | ZPLD1 | IR | 1 | MIIKNSLSL | FSILSVQRFK SILSVQRFK |
| chr10:67956976\|67959807:- | HERC4 | IR | 1 | FLFNKVLADI FLFNKVLA | STRKCTFLFNK |
| chr6:89889425\|89894453:+ | GJA10 | IGR | 1 | NMLLELSSI MLLELSSIM KIMHQEQLV ALWDPHPL ALWDPHPLQT | RTFIYYEK RVLPAWAHFHR ASRTFIYYEK HASRTFIYY HASRTFIYYEK |
| chr12:113410108\|113427962:+ | SDSL | IGR | 1 | VLCGTSCHL | HTHTIHMHR |
| chr12:21638470\|21638577:- | LDHB | IR | 1 | . | RSDMLLFEGK TQQFCFALK QQFCFALKFLK MTQQFCFALK |
| chr3:183039135\|183039313:- | MCCC1 | IR | 1 | FQSPNTYLL IYLFSKEVLRL YLFSKEVL YLFSKEVLRLT YLFSKEVLRL | . |
| chrX:49793710\|49830399:+ | PAGE4 | IGR | 1 | LVAAMSARV GMWTGHFCGL | WTGHFCGLSK |
| chr19:48345354\|48354582:- | TMEM143 | IR | 1 | . | STEEETSNK |
| chr7:139542748\|139544231:+ | CLEC2L | IR | 1 | TQVEWMFTI | . |
| chr17:42088295\|42088425:+ | ZNF385C | IR | 1 | . | NHTMAETRVK HTMAETRVK VNHTMAETRVK RVNHTMAETR |
| chr6:75530550\|75535343:- | FILIP1 | IGR | 1 | TMPRSFWKA | TQNTMPRSFWK TVAVVGMRSR VTVAVVGMR NTMPRSFWK QNTMPRSFWK |
| chr7:111561923\|111593575:- | IMMP2L | IGR | 1 | TILGVLERL GLLSKGFGQI | KITTYPFSPR TTYPFSPRK ITTYPFSPR ERLTLSGLLSK TTYPFSPRKS RLTLSGLLSK TTYPFSPR KITTYPFSPRK LTLSGLLSK GTILGVLER ITTYPFSPRK MGTILGVLER |
| chr1:173676391\|173691113:- | ANKRD45 | IR | 1 | RIWKLFVFL KLFVFLELM KLFVFLEL | . |

**Supplemental Table S2. NeoTSTs of Hep53.4 cell line.**

| Junction_38 | Gene | neopep_range | netmhcpan1 | netmhcpan2 |
| --- | --- | --- | --- | --- |
| chr1:135154795\|135155221:+ | Arl8a | 172>197 | CYSISCKEKDNIGAWLGHHPTVAYSTLKVTEKLRLWLFF | DREICCYSISCKEKDNIGAWLGHHPTVAYSTLKVTEKLRLWLFF |
| chr17:14677093\|14677360:- | Thbs2 | 1>59 | MYSCERSSSFETCWPVRTELARMLELLVHLMSWGKEQDAKHLALHLSWDRRPHLPLFSQDNCPKLPNSGQE | MYSCERSSSFETCWPVRTELARMLELLVHLMSWGKEQDAKHLALHLSWDRRPHLPLFSQDNCPKLPNSGQEDFDKDG |
| chr6:137519516\|137519835:- | Eps8 | 1>36 | MWGWGVTSSPGCGDGSVGIVLATPACRAESHSPCPVEKPRQYHEQEET | MWGWGVTSSPGCGDGSVGIVLATPACRAESHSPCPVEKPRQYHEQEETPEMMAA |
| chr12:84310958\|84311137:+ | Ptgr2 | 327>348 | KETMAKGLENMGEAQGTSVSGNTVLLSMQPGGMPVAFQSMMTGGNV | EGKLKVKETMAKGLENMGEAQGTSVSGNTVLLSMQPGGMPVAFQSMMTGGNVGKQIVC |
| chr19:5819850\|5856585:- | Frmd8 | 451>496 | GRTLARPHHWGQQGEHGHSRSLSHRSLIREDNEQASLRWRSGWQHPHRHTLRELLGCS | EFPVRAGRTLARPHHWGQQGEHGHSRSLSHRSLIREDNEQASLRWRSGWQHPHRHTLRELLGCS |

**Supplemental Table S3. MHCPan I prediction of neoTSTs of hep53.4.**

| MHC | Peptide | Core | Identity | Aff(nM) | BindLevel |
| --- | --- | --- | --- | --- | --- |
| H-2-Kk | FETCWPVRTEL | FETPVRTEL | chr17:14677093\|14677360:- | 104.06 | <=WB |
| H-2-Kk | TELARMLELL | TELARLELL | chr17:14677093\|14677360:- | 74.02 | <=WB |
| H-2-Kq | FETCWPVRTEL | FEWPVRTEL | chr17:14677093\|14677360:- | 440.62 | <=WB |
| H-2-Kq | TELARMLELL | TELARLELL | chr17:14677093\|14677360:- | 217.24 | <=WB |
| H-2-Kb | RMLELLVHL | RMLELLVHL | chr17:14677093\|14677360:- | 183.81 | <=SB |
| H-2-Kb | LSWDRRPHL | LSWDRRPHL | chr17:14677093\|14677360:- | 115.35 | <=SB |
| H-2-Kk | TELARMLEL | TELARMLEL | chr17:14677093\|14677360:- | 55.51 | <=SB |
| H-2-Kk | LELLVHLM | LEL-LVHLM | chr17:14677093\|14677360:- | 40.59 | <=SB |
| H-2-Kq | TELARMLEL | TELARMLEL | chr17:14677093\|14677360:- | 168.23 | <=SB |
| H-2-Kq | LELLVHLM | LEL-LVHLM | chr17:14677093\|14677360:- | 160.92 | <=SB |
| H-2-Kk | AESHSPCPV | AESHSPCPV | chr6:137519516\|137519835:- | 187.23 | <=WB |
| H-2-Kk | TEKLRLWLF | TEKLRLWLF | chr1:135154795\|135155221:+ | 395.24 | <=WB |
| H-2-Kk | TEKLRLWL | TEK-LRLWL | chr1:135154795\|135155221:+ | 131.64 | <=WB |
| H-2-Kq | TEKLRLWL | TEKLRLW-L | chr1:135154795\|135155221:+ | 499.88 | <=WB |
| H-2-Dq | HPTVAYSTL | HPTVAYSTL | chr1:135154795\|135155221:+ | 294.26 | <=SB |
| H-2-Kb | VAYSTLKV | VAYS-TLKV | chr1:135154795\|135155221:+ | 159.33 | <=SB |
| H-2-Kq | TEKLRLWLF | TEKLRLWLF | chr1:135154795\|135155221:+ | 289.73 | <=SB |
| H-2-Ld | HPTVAYSTL | HPTVAYSTL | chr1:135154795\|135155221:+ | 297.36 | <=SB |
| H-2-Db | TSVSGNTVLL | TSVSNTVLL | chr12:84310958\|84311137:+ | 483.55 | <=SB |
| H-2-Dq | SMQPGGMPVAF | SPGGMPVAF | chr12:84310958\|84311137:+ | 375.49 | <=SB |
| H-2-Dq | MQPGGMPVAF | MPGGMPVAF | chr12:84310958\|84311137:+ | 87.16 | <=SB |
| H-2-Dq | QPGGMPVAF | QPGGMPVAF | chr12:84310958\|84311137:+ | 123.23 | <=SB |
| H-2-Dq | MPVAFQSMM | MPVAFQSMM | chr12:84310958\|84311137:+ | 136.11 | <=SB |
| H-2-Kk | GEHGHSRSL | GEHGHSRSL | chr19:5819850\|5856585:- | 258.39 | <=SB |
| H-2-Kk | WQHPHRHTL | WQHPHRHTL | chr19:5819850\|5856585:- | 457.8 | <=SB |
| H-2-Ld | QPGGMPVAF | QPGGMPVAF | chr12:84310958\|84311137:+ | 461.78 | <=SB |
| H-2-Ld | MPVAFQSMM | MPVAFQSMM | chr12:84310958\|84311137:+ | 157.57 | <=SB |
| H-2-Lq | MPVAFQSMM | MPVAFQSMM | chr12:84310958\|84311137:+ | 164 | <=SB |

**Supplemental Table S4. MHCPan II prediction of neoTSTs of hep53.4.**

| MHC | Peptide | Core | Identity | Affinity(nM) | BindLevel |
| --- | --- | --- | --- | --- | --- |
| H-2-IAb | AWLGHHPTVAYSTLK | GHHPTVAYS | chr1:135154795\|135155221:+ | 434.72 | <=WB |
| H-2-IAb | NIGAWLGHHPTVAYST | WLGHHPTVA | chr1:135154795\|135155221:+ | 464.11 | <=WB |
| H-2-IAb | GAWLGHHPTVAYSTLK | GHHPTVAYS | chr1:135154795\|135155221:+ | 330.43 | <=WB |
| H-2-IAu | HHPTVAYSTLKVTEK | VAYSTLKVT | chr1:135154795\|135155221:+ | 309.99 | <=WB |
| H-2-IAu | GHHPTVAYSTLKVTEK | VAYSTLKVT | chr1:135154795\|135155221:+ | 257.77 | <=WB |
| H-2-IAu | LGHHPTVAYSTLKVTEK | VAYSTLKVT | chr1:135154795\|135155221:+ | 292.59 | <=WB |
| H-2-IAu | GIVLATPACRAESH | VLATPACRA | chr6:137519516\|137519835:- | 434.84 | <=WB |
| H-2-IAu | VGIVLATPACRAESH | VLATPACRA | chr6:137519516\|137519835:- | 236.33 | <=WB |
| H-2-IAu | SVGIVLATPACRAESH | VLATPACRA | chr6:137519516\|137519835:- | 256.43 | <=WB |
| H-2-IEk | SVGIVLATPACRAES | IVLATPACR | chr6:137519516\|137519835:- | 36.9 | <=WB |
| H-2-IAu | GTSVSGNTVLLSMQP | VSGNTVLLS | chr12:84310958\|84311137:+ | 332.53 | <=WB |
| H-2-IAu | QGTSVSGNTVLLSMQP | VSGNTVLLS | chr12:84310958\|84311137:+ | 412.01 | <=WB |
| H-2-IAu | AQGTSVSGNTVLLSMQP | VSGNTVLLS | chr12:84310958\|84311137:+ | 445.4 | <=WB |

**Supplemental Table S5. NeoMuts of Hep53.4 cell line.**

| Variant | peptide | gene | DNA_change | Protein_change | Protein_change |
| --- | --- | --- | --- | --- | --- |
| chr16:20662660-20662660 | ILGKSHYVLYGL**I**AAMQPRMLVTFD | Psmd2 | c.G2464A | p.V822I | p.V822I |
| chr17:35052459-35052459 | LFVKAGSDGAKIWNCPFSQRLFMVL | Clic1 | c.G64T | p.G22W | p.G22W |
| chr13:58393218-58393218 | ITITGTQDQIQNTQYLLQNSVKQYS | Hnrnpk | c.G1132A | p.A378T | p.A378T |
| chr13:49688625-49688625 | AEYNKQCRAIVM**S**YSAEWKSTVTRL | Iars | c.A378C | p.R126S | p.R126S |
| chr15:89160395-89160395 | LILPLVMVPMVF**S**IVVSIYCYWRKS | Plxnb2 | c.T3647G | p.I1216S | p.I1216S |
| chr4:52481682-52481682 | ALNIAWQKVNKDLGSIFSTLLPGAN | Smc2 | c.T3121C | p.F1041L | p.F1041L |
| chr4:101163681-101163681 | RQVSHKHIVYLY**V**VCVRDVENIMVE | Jak1 | c.G1961T | p.G654V | p.G654V |
| chr19:4287645-4287645 | GKDCIVHGYMSK**I**GNPFLTQWQRRY | Grk2 | c.G1704C | p.M568I | p.M568I |
| chr10:81046280-81046280 | QAGQQFAQQMQQLNPEFVEQIRSQV | Sgta | c.A863T | p.Q288L | p.Q288L |
| chr6:17535069-17535069 | ETSSFSYREDPV**L**YEIHPTKSFISG | Met | c.G2227C | p.V743L | p.V743L |
| chr8:123997362-123997362 | GQMSNVLSVQFM**S**CNLLLVTGITQE | Taf5l | c.G1717T | p.A573S | p.A573S |
| chr18:67552585-67552585 | EKRSISAIRSYQ**Y**VMKICAAHLPTE | Spire1 | c.G118T | p.D40Y | p.D40Y |
| chr5:134263645-134263645 | PEGIPFRRPSTY**V**IPRLERILLAKE | Gtf2i | c.G989T | p.G330V | p.G330V |
| chr6:119990128-119990128 | LEMATSEYPYSE**W**QNAAQIYRRVTS | Wnk1 | c.C1275G | p.C425W | p.C425W |
| chr14:24164486-24164486 | DKKQAIKALLNG**D**GAINMVVRRRKS | Dlg5 | c.G1035T | p.E345D | p.E345D |
| chr9:44329125-44329125 | LVISASIIVFNL**L**ELEGDYRDDHIF | Dpagt1 | c.G637T | p.V213L | p.V213L |
| chr4:129341521-129341521 | AWGDTLEEAFEQ**S**AMAMFGYMTDTG | zbtb8os | c.G167C | p.C56S | p.C56S |
| chr5:76230281-76230281 | VQLSSGNSNIQQ**V**TPVNMQGQVVPA | Clock | c.C1771G | p.L591V | p.L591V |
| chr1:60082564-60082564 | LWSDAEEICSAS**L**DHTIRVWDVESGG | Wdr12 | c.G827T | p.W276L | p.W276L |
| chr5:150662320-150662320 | ARDETAALLNSAV**L**GAAPLFVPPAD | N4bp2l2 | c.G194T | p.R65L | p.R65L |

**Supplemental Table S6. GSEA reault of neoTSTs group.**

| NAME | SIZE | NES | FDR q-val | FWER p-val | RANK AT MAX |
| --- | --- | --- | --- | --- | --- |
| ACTIVATED CD8 T CELL | 24 | 1.8377206 | 0 | 0 | 877 |
| PDL1_EXPRESSION_AND_PD1_CHECKPOINT_PATHWAY_IN_CANCER | 86 | 1.7832985 | 0.001216154 | 0.002 | 2013 |
| ANTIGEN_PROCESSING_AND_PRESENTATION | 104 | 1.7170447 | 0.002316291 | 0.006 | 3401 |
| TYPE 1 T HELPER CELL | 66 | 1.6919662 | 0.002599118 | 0.009 | 3045 |
| APCAF | 34 | 1.6725534 | 0.003450698 | 0.015 | 1573 |
| ACTIVATED B CELL | 17 | 1.6635748 | 0.003642654 | 0.019 | 2315 |
| INFLAMMATORY RESPONSE TO ANTIGEN STIMULUS | 64 | 1.6474379 | 0.004354524 | 0.026 | 2081 |
| CYTOKINE PRODUCTION INVOLVED IN INFLAMMATION | 61 | 1.6185517 | 0.008094206 | 0.055 | 2948 |
| ACTIVATED CD4 T CELL | 23 | 1.5783647 | 0.014355553 | 0.108 | 2095 |
| TLR SIGNLING | 73 | 1.5347598 | 0.028818756 | 0.222 | 3655 |
| REGULATORY T CELL | 18 | 1.4991313 | 0.04254338 | 0.338 | 1980 |
| ACTIVATED DENDRITIC CELL | 27 | 1.4660207 | 0.060379066 | 0.478 | 3309 |
| IMMATURE B CELL | 15 | 1.4456054 | 0.07015623 | 0.559 | 3824 |
| MDSC | 17 | 1.4424635 | 0.067768574 | 0.573 | 2569 |
| NATURAL KILLER CELL | 30 | 1.4069973 | 0.09005129 | 0.697 | 3370 |
| EFFECTOR MEMEORY CD8 T CELL | 17 | 1.360407 | 0.13216347 | 0.845 | 3403 |
| MACROPHAGE | 24 | 1.352686 | 0.1335162 | 0.858 | 3314 |
| T FOLLICULAR HELPER CELL | 27 | 1.3434591 | 0.13480604 | 0.876 | 3104 |
| NATURAL KILLER T CELL | 34 | 1.3379565 | 0.13302764 | 0.885 | 2596 |
| TYPE 17 T HELPER CELL | 18 | 1.3193766 | 0.14727078 | 0.915 | 1258 |
| IMMATURE DENDRITIC CELL | 22 | 1.3088251 | 0.15321726 | 0.935 | 890 |
| TYPE 2 T HELPER CELL | 25 | 1.2712604 | 0.19584487 | 0.975 | 2704 |
| EFFECTOR MEMEORY CD4 T CELL | 23 | 1.1238425 | 0.4230956 | 1 | 2083 |
| MONOCYTE | 17 | 1.0957549 | 0.45632014 | 1 | 1312 |
| CD56BRIGHT NATURAL KILLER CELL | 31 | 1.0780623 | 0.47087252 | 1 | 1262 |
| CENTRAL MEMORY CD8 T CELL | 20 | 1.0733435 | 0.46114072 | 1 | 2973 |
| RESPONSE TO TYPE-I IFN | 67 | 1.0552324 | 0.4754363 | 1 | 4273 |
| PLASMACYTOID DENDRITIC CELL | 27 | 0.8611819 | 0.7762983 | 1 | 3256 |
| RGI-I | 22 | 0.85977995 | 0.75136566 | 1 | 3877 |
| GAMMA DELTA T CELL | 21 | 0.76508284 | 0.8490017 | 1 | 3216 |
| CENTRAL MEMORY CD4 T CELL | 24 | 0.7143816 | 0.87379783 | 1 | 1524 |

**Supplemental Table S7. GSEA reault of neoMuts group.**

| NAME | SIZE | NES | FDR q-val | FWER p-val | RANK AT MAX |
| --- | --- | --- | --- | --- | --- |
| ACTIVATED CD8 T CELL | 24 | 1.8772501 | 0 | 0 | 1263 |
| PDL1_EXPRESSION_AND_PD1_CHECKPOINT_PATHWAY_IN_CANCER | 86 | 1.6999031 | 0.010093175 | 0.017 | 2645 |
| TYPE 1 T HELPER CELL | 66 | 1.6686145 | 0.009944616 | 0.025 | 1647 |
| ANTIGEN_PROCESSING_AND_PRESENTATION | 103 | 1.5537099 | 0.05724932 | 0.176 | 3071 |
| CYTOKINE PRODUCTION INVOLVED IN INFLAMMATION | 62 | 1.5406934 | 0.054515563 | 0.208 | 3240 |
| TYPE 2 T HELPER CELL | 25 | 1.5293505 | 0.05279326 | 0.241 | 1750 |
| APCAF | 34 | 1.5196139 | 0.051728956 | 0.272 | 2489 |
| TLR SIGNLING | 73 | 1.4674938 | 0.09210502 | 0.468 | 3968 |
| INFLAMMATORY RESPONSE TO ANTIGEN STIMULUS | 64 | 1.4431314 | 0.10995279 | 0.572 | 2642 |
| NATURAL KILLER T CELL | 34 | 1.4148184 | 0.13087462 | 0.667 | 2533 |
| EFFECTOR MEMEORY CD8 T CELL | 17 | 1.4076517 | 0.12915556 | 0.697 | 3547 |
| MDSC | 17 | 1.39467 | 0.13437985 | 0.741 | 2635 |
| ACTIVATED CD4 T CELL | 23 | 1.387653 | 0.13380373 | 0.771 | 2910 |
| IMMATURE B CELL | 15 | 1.3872043 | 0.12508187 | 0.772 | 3868 |
| T FOLLICULAR HELPER CELL | 27 | 1.3858055 | 0.117752336 | 0.773 | 3379 |
| TYPE 17 T HELPER CELL | 18 | 1.3218039 | 0.18692535 | 0.929 | 747 |
| MACROPHAGE | 24 | 1.3177263 | 0.18148452 | 0.935 | 3691 |
| NATURAL KILLER CELL | 30 | 1.314843 | 0.17506549 | 0.942 | 3651 |
| ACTIVATED B CELL | 17 | 1.3006634 | 0.18382937 | 0.962 | 2824 |
| CD56BRIGHT NATURAL KILLER CELL | 31 | 1.1996332 | 0.33360487 | 0.998 | 1532 |
| REGULATORY T CELL | 18 | 1.1682707 | 0.37722257 | 0.999 | 3280 |
| RGI-I | 22 | 1.0421963 | 0.60402375 | 1 | 3976 |
| EFFECTOR MEMEORY CD4 T CELL | 23 | 0.9780412 | 0.70885426 | 1 | 4430 |
| ACTIVATED DENDRITIC CELL | 26 | 0.9456861 | 0.744784 | 1 | 3526 |
| IMMATURE DENDRITIC CELL | 22 | 0.9116771 | 0.77812904 | 1 | 1582 |
| CENTRAL MEMORY CD8 T CELL | 20 | 0.9097418 | 0.75173277 | 1 | 1774 |
| CENTRAL MEMORY CD4 T CELL | 24 | 0.8945877 | 0.7505276 | 1 | 45 |
| MONOCYTE | 17 | 0.8706512 | 0.75914806 | 1 | 4379 |
| RESPONSE TO TYPE-I IFN | 67 | 0.8338143 | 0.7852603 | 1 | 4607 |
| GAMMA DELTA T CELL | 22 | 0.79678714 | 0.8097201 | 1 | 2620 |
| PLASMACYTOID DENDRITIC CELL | 27 | 0.70657915 | 0.8828687 | 1 | 2765 |

**Reference**

1. Dobin A, Davis CA, Schlesinger F, Drenkow J, Zaleski C, Jha S, et al. STAR: ultrafast universal RNA-seq aligner. Bioinformatics. 2013;29(1):15-21.

2. Pertea M, Pertea GM, Antonescu CM, Chang TC, Mendell JT, Salzberg SL. StringTie enables improved reconstruction of a transcriptome from RNA-seq reads. Nat Biotechnol. 2015;33(3):290-5.

3. Zhao J, Li Q, Li Y, He X, Zheng Q, Huang S. ASJA: A Program for Assembling Splice Junctions Analysis. Comput Struct Biotechnol J. 2019;17:1143-50.

4. Wang L, Park HJ, Dasari S, Wang S, Kocher JP, Li W. CPAT: Coding-Potential Assessment Tool using an alignment-free logistic regression model. Nucleic Acids Res. 2013;41(6):e74.

5. Cock PJ, Antao T, Chang JT, Chapman BA, Cox CJ, Dalke A, et al. Biopython: freely available Python tools for computational molecular biology and bioinformatics. Bioinformatics. 2009;25(11):1422-3.

6. Orenbuch R, Filip I, Comito D, Shaman J, Pe'er I, Rabadan R. arcasHLA: high-resolution HLA typing from RNAseq. Bioinformatics. 2020;36(1):33-40.

7. Reynisson B, Alvarez B, Paul S, Peters B, Nielsen M. NetMHCpan-4.1 and NetMHCIIpan-4.0: improved predictions of MHC antigen presentation by concurrent motif deconvolution and integration of MS MHC eluted ligand data. Nucleic Acids Res. 2020;48(W1):W449-w54.

8. Kong AT, Leprevost FV, Avtonomov DM, Mellacheruvu D, Nesvizhskii AI. MSFragger: ultrafast and comprehensive peptide identification in mass spectrometry-based proteomics. Nat Methods. 2017;14(5):513-20.

9. Szolek A, Schubert B, Mohr C, Sturm M, Feldhahn M, Kohlbacher O. OptiType: precision HLA typing from next-generation sequencing data. Bioinformatics. 2014;30(23):3310-6.

10. Wu J, Chen W, Zhou Y, Chi Y, Hua X, Wu J, et al. TSNAdb v2.0: The Updated Version of Tumor-specific Neoantigen Database. Genomics Proteomics Bioinformatics. 2023;21(2):259-66.

11. Yu H, Wen Y, Yu W, Lu L, Yang Y, Liu C, et al. Optimized circular RNA vaccines for superior cancer immunotherapy. Theranostics. 2025;15(4):1420-38.

12. Yu H, Yang Y, Lin P, Liu C, Wen Y, Huang Z, et al. An engineered linear cap-independent mRNA vaccine with intrinsic adjuvanticity induces potent anti-tumor immunity in mice. Nat Commun. 2026;17(1).
